# Innate and Adaptive Immunity during SARS-CoV-2 Infection: Biomolecular Cellular Markers and Mechanisms

**DOI:** 10.3390/vaccines11020408

**Published:** 2023-02-10

**Authors:** Brent Brown, Vanshika Ojha, Ingo Fricke, Suhaila A Al-Sheboul, Chinua Imarogbe, Tanya Gravier, Michael Green, Lori Peterson, Ivoyl P. Koutsaroff, Ayça Demir, Jonatane Andrieu, Chiuan Yee Leow, Chiuan Herng Leow

**Affiliations:** 1 Academic Researcher, London NW7 4AU, UK; 2 Independent Researcher, Ayodhya 224001, India; 3 Independent Immunologist and Researcher, 311995 Lamspringe, Germany; 4 Department of Medical Laboratory Sciences, Faculty of Applied Medical Sciences, Jordan University of Science and Technology, Irbid 22110, Jordan; 5 Department of Medical Microbiology, International School of Medicine, Medipol University-Istanbul, Istanbul 34810, Turkey; 6 UKHSA, Rosalind Franklin Laboratory, UK; 7 Independent Researcher, MPH, San Francisco, CA 94131, USA; 8 Independent Researcher, Perth 6065, Australia; 9 Senior Epidemiologist, Murray, KY 42071, USA; 10 Technology Freelancer, Otsu, Osaka 520-0817, Japan; 11 Faculty of Medicine, Afyonkarahisar University, Istanbul 03030, Turkey; 12 Faculté de Médecine, Aix–Marseille University, 13005 Marseille, France; 13 School of Pharmaceutical Sciences, Universiti Sains Malaysia, USM, Penang 11800, Malaysia; 14 Institute for Research in Molecular Medicine, (INFORMM), Universiti Sains Malaysia, USM, Penang 11800, Malaysia

**Keywords:** COVID-19, B-cells, neutrophils, dendritic cells, T-cells, NK-cells, monocytes, macrophages, innate, adaptive, cytokines, chemokines, adhesion molecules, antibody, cluster of differentiation, receptors, proteins, SARS-CoV-2, serology

## Abstract

The coronavirus 2019 (COVID-19) pandemic was caused by a positive sense single-stranded RNA (ssRNA) severe acute respiratory syndrome coronavirus 2 (SARS-CoV-2). However, other human coronaviruses (hCoVs) exist. Historical pandemics include smallpox and influenza, with efficacious therapeutics utilized to reduce overall disease burden through effectively targeting a competent host immune system response. The immune system is composed of primary/secondary lymphoid structures with initially eight types of immune cell types, and many other subtypes, traversing cell membranes utilizing cell signaling cascades that contribute towards clearance of pathogenic proteins. Other proteins discussed include cluster of differentiation (CD) markers, major histocompatibility complexes (MHC), pleiotropic interleukins (IL), and chemokines (CXC). The historical concepts of host immunity are the innate and adaptive immune systems. The adaptive immune system is represented by T cells, B cells, and antibodies. The innate immune system is represented by macrophages, neutrophils, dendritic cells, and the complement system. Other viruses can affect and regulate cell cycle progression for example, in cancers that include human papillomavirus (HPV: cervical carcinoma), Epstein–Barr virus (EBV: lymphoma), Hepatitis B and C (HB/HC: hepatocellular carcinoma) and human T cell Leukemia Virus-1 (T cell leukemia). Bacterial infections also increase the risk of developing cancer (e.g., *Helicobacter pylori*). Viral and bacterial factors can cause both morbidity and mortality alongside being transmitted within clinical and community settings through affecting a host immune response. Therefore, it is appropriate to contextualize advances in single cell sequencing in conjunction with other laboratory techniques allowing insights into immune cell characterization. These developments offer improved clarity and understanding that overlap with autoimmune conditions that could be affected by innate B cells (B1^+^ or marginal zone cells) or adaptive T cell responses to SARS-CoV-2 infection and other pathologies. Thus, this review starts with an introduction into host respiratory infection before examining invaluable cellular messenger proteins and then individual immune cell markers.

## 1. Introduction

### 1.1. Overview

The causal virion SARS-CoV-2 of the COVID-19 pandemic contains more than four immunogenic proteins composed of spike (S protein), nucleocapsid (N protein), envelope (E protein), and membrane (M protein) and associated subunits with accessory proteins [1,2]. Current therapeutic development occurred before/after March 2020 when the World Health Organization (WHO) declared a pandemic; SARS-CoV-2 is known to spread between animals and between generations [3]. It is thought that around 15% of COVID-19 disease mortality could be due to pneumonia or acquired respiratory distress syndrome (ARDS) [4]. Current vaccine immunogens were largely developed as pre-fusion S protein derivatives with newer candidates progressing through clinical trials with a goal to reduce chronic COVID-19 disease burden within populations as research continues. The SARS-CoV-2 genome is approximately 30 kilobases encoding 9860 amino acids and defined by open reading frames (ORF) and non-structural proteins (NSP) required for viral propagation within all animal hosts [5]. The SARS-CoV-2 genome hosts 16 ORFs that encode 29 proteins required for viral propagation and host immune response inhibition. For example, ORF1a and ORF1ab encode polypeptides cleaved into 16 NSPs. Molecular testing methods (e.g., PCR) are commonly used diagnostic tools that allow analysis and detection of specific RNA sequences within samples. SARS-CoV-2 infects cells via respiratory pathways and type II pneumocytes (ATII) using angiotensin converting enzyme 2 (ACE-2) as the predominant receptor for entry [6]. Disruption and infection of ATII cells expressing ACE2 occurs through phospholipid membranes. Other receptors expressed on all leukocytes, platelets, and endothelial cells include varying clusters of differentiation markers (CD), for example CD3, CD4, and CD19, among others. Some are also currently implicated in initial SARS-CoV-2 cellular entry that include type II transmembrane protease (TMPRSS2), asialoglycoprotein receptor-1 (ASGR1) and kringle containing transmembrane protein 1 (KREMEN1), dipeptidyl peptidase 4 (DPP4), neuropilin (NRP1), CD147 and vimentin [7,8,9,10,11,12]. Therefore, because of cellular infection, immune cells regulate and develop within primary lymph organs (e.g., bone marrow and thymus), but also through a network of secondary lymph organs (e.g., tonsils and others) utilizing lymph nodes (LNs) and cellular membranes that allow cellular permeability and lymphocyte migration to process infectious pathogenic proteins at all barriers through the nervous, digestive, endocrine, respiratory, circulatory, muscular, and skeletal systems. Technological advancement since 2017 has also allowed greater phenotypic analysis and, therefore, it is clearer now that SARS-CoV-2 proteins have different host roles. Analyses have confirmed that M protein is vital for assembling, S protein is for cellular receptor entry, and N and E proteins appear to be potential pore forming proteins [13,14]. Single cell RNA sequencing (scRNA-Seq), spectral flow cytometry (FACS), and mass cytometry (CyTOF) can detect markers enabling phenotypic analysis of all immune cell subsets [15,16,17,18]. It is noteworthy that antibody proteins involved in testing for SARS-CoV-2 infections have variable factors and human antibody concentrations. These are measured by predominant monoclonal antibody diagnostics for which many scales exist that undergo validation regardless of manufacturer. For example, institutions measure concentrations in sera utilizing several assays that measure binding antibodies (BAU/mL), others measure neutralizing antibodies (nAb in IU/mL), and others measure concentration (ng/mL) as standardization occurs to ensure consistency on a global scale (Supplementary Materials and Data S1–S5) [19,20]. Therefore, in this paper, we review current existing laboratory and clinical studies that have measured and illustrated statistical significance to illustrate the changing immune cellular maturation environment, to hypothesize that many of the inflammatory effects seen with SARS-CoV-2 infection can be dysregulated adaptive immune responses. Multiple bacterial, viral, and fungal pathologies have both immunological and genetic variability factors affected by cell cycle regulation and antigen presentation factors. These affect multiple pathologies, and risk is therefore affected by genetic susceptibility factors. This includes genes that encode proteins expressed on immune cell membranes, such as human leukocyte antigens (HLA), referred to as the major histocompatibility complex (MHC). These present short protein (peptide) fragments to the immune system cells and localized cellular markers affected by other mediator proteins, as we discuss below. The immune cells discussed in this paper provide overall context with further cell characterization through interaction of B cells, T cells, and each of the other four cell subtypes discussed below, classified by specifically by cluster of differentiation (CD) proteins expressed on cell surfaces reliant on cytokine and chemokines acting as immune cell signals affected by external pathogens.

### 1.2. Current SARS-CoV-2 Vaccine Immunogen Responses 

Current vaccine antigens or viral antigens prime the immune system to recognize pathogenic proteins via epitopes that can mutate, thereby affecting immune cell recognition through B cell and T cell receptors (BCR/TCR). Prevention of chronic COVID-19 disease to pre-Omicron variants was estimated in these ratios within current vaccine immunogens, developed by Pfizer/BioNTech, Astra Zeneca, Sinopharm, and Novavax: BNT162b2: 95.3%, AZD1222: 70.4%, BBIBP-CorV: 79%. Immunogen development indicates NVX-CoV2373 at 72% when screened against Omicron BA.1 and BA4/BA5 [21]. Additional risk reduction of COVID-19 disease was estimated at 86%. Population studies show variable SARS-CoV-2 protein antibody responses (76%:24% response/non-response). Estimated production of functional antibodies to S protein immunogens currently spans 6 months to 1.5 years. SARS-CoV-2 S protein mutations are now well documented in other studies to ascertain potential epitopes that affect immune response [22]. Functional cellular T cell responses, either helper (T_H_) or cytotoxic (T_C_), are also suggestive of CD4^+^:CD8^+^ activity occurrence in the ratio 96%:54% in COVID-19 disease [21]. Compared to other respiratory viruses such as influenza, the SARS-CoV-2 S protein possesses higher mutational rates within the spike/ACE2 interface, and the emergence of Omicron variants support this, denoted by BA1, BA2, BA2.75, BA4, BA5, BQ1, and XBB [23]. Fortunately, technology and laboratory techniques exist that facilitate accurate cell profiling and allow comparisons of relevant immune cells. Therefore, in this paper, we will discuss route of infection, predominant cytokine and protein markers, and, finally, individual cellular responses of predominant immune cell lineages in order of B cells, neutrophils, monocytes/macrophages/dendritic cells (DC), natural killer (NK cells), and T cell subtypes. 

### 1.3. Respiratory Microenvironment

**Figure 1:**
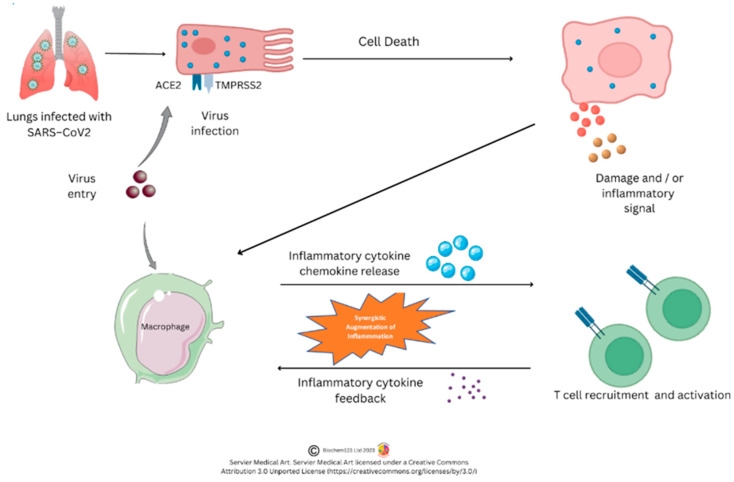
Overview of SARS-CoV-2 immune cell interactions.

Respiratory tract organs affected are nose, throat, larynx, trachea, bronchi, and lungs exposed to external antigens composed of surface epithelial cell layers. An adult human lung surface area contains approximately 700 million alveoli, with a surface area of 70 m^2^ and a diameter of between 200 μm and 500 μm, covered by capillaries. Within this defined alveolar layer are ciliated type I pneumocytes (ATI) cells, as well as type II pneumocyte (ATII) cells and alveolar Mϕ (AMϕ) that regulate respiration, secretion of surfactant, and immune cell regulation, respectively, alongside goblet cells, basal cells, and other cell types [24]. Early studies (n = 7) in chronic SARS-CoV-2 induced disease show direct infection of ATII cells through the glycocalyx and surfactant layer, thereby compromising homeostatic barriers and valve functions through increased pressure of inhaled O_2_ or exhaled CO_2_ within nanobubbles across cell membranes where CO_2_ is produced through the tricarboxylic acid (TCA) cycle [25]. The glycocalyx layer is known to contain an abundance of proteins that affect vascular function (e.g., syndecans) that can be degraded and affect the vasculature, such as matrix metalloproteinases (MMP), heparanase, and hyaluronidase, through the action of cytokines (IL-1β and others) [26,27]. This process of respiration is dependent on membrane thickness and gas solubility of O_2_, N_2_, and CO_2_ nanobubbles [28] (see Figure 1).

Type II pneumocyte (ATII) cells, responsible for epithelial cell repair and renewal, are specialized secretory cells that produce a surfactant consisting of 90% lipids (mainly saturated phospholipids) and 10% pulmonary surfactant proteins (SP) that contribute to the surface tensile maintenance utilizing dipalmitoylphosphatidylcholine (DPPC), and other SPs that are synthesized in the endoplasmic reticulum of ATII cells. SP-A, SP-B, SP-C, SP-D, SP-B, and SP-C are hydrophobic proteins with SP-A and SP-D possessing intermixed hydrophilic components that participate in pulmonary host defense. These proteins act as valves, resulting in dysregulation of pathogen. Specifically, SP-A and SP-D valve-like activities disrupted on pathogen clearance would result in dampened antigen presentation or allergy-induced immune function of cells, utilizing a network of cytokine processes that regulate immune cell activity and development [29]. Molecular studies further elucidated that gene changes that would produce SP-B and SP-C proteins occurred with downregulation of a transcription factor (ETV5) specifically required for ATII cell maintenance alongside a cellular chloride channel (CLIC5) [30,31].

### 1.4. Cytokine and Serum Proteins during SARS-CoV-2 Infection

Serum protein elevation, documented as a “cytokine storm” that is elevated or dysfunctional in SARS-CoV-2 induced chronic COVID-19 disease, occurs in many other pathologies [32]. Cytokines are a group of short-lived proteins released by various cells acting as intercellular messengers. Cytokine synthesis and secretory mechanisms include release from lysosomes, shedding of vesicles from plasma membranes, and release from plasma membranes. Many studies document these, which are not the main topic of this review. In comparison, in influenza (genus Influenza A/B/C/D) infection, the cytokines IL-1β, IL-4, IL-5, IL-6, IL-10, IL-12, IL-13, TNF-α, and IFN-γ are relevant to immune cell interaction, as outlined below in figures. During SARS-CoV-2 infection induced COVID-19 disease, other proteins have been considered, which include transforming and vascular endothelial cellular growth factors (TGF-β/VEGF) in conjunction with specific MMPs (MMP2, MMP3, MMP9) [33,34,35,36]. These represent tissue re-modeling proteins with specific chemotactic factors also required to direct leukocyte chemotaxis between germinal center lymph systems (GC) and throughout the body [34,35,36]. Relevant chemokines considered below include CXCL10 (IP-10), CCL2 (MCP-1), CCL3 (MIP1-α), and CCL11 [33,34,35,36,37]. However, prior to the 2020 pandemic, in a related coronavirus (MERS-CoV) causing Middle East Respiratory Syndrome (MERS), the cytokine proteins IL-1β, IL-6 and IL-8 were highlighted as key to host response, whereas, in infection, CXCL10 and other pleiotropic chemokines are further investigated that utilize CXCR3 expressed on Mϕ, T cells, DCs, and both NK/B cells [38,39,40]. The above cytokines and chemokines are, therefore, all innate/adaptive regulators that contribute towards infection control and regulation within blood serum. Studies indicate COVID-19-associated coagulopathy (CAC) is a causal factor in chronic disease with complexes formed between innate immune cells affecting coagulation and fibrinolytic processes through unknown mechanisms. Categorization of COVID-19 has, therefore, occurred in vascular endothelial cell dysfunction, hyper-inflammatory response, and hypercoagulability, documenting this aspect of SARS-CoV-2 induced pathology with resulting serum elevation in plasma levels of D-dimer, C-reactive protein, P-selectin, and fibrinogen [41]. More recently, in a yet-to-be-reviewed pre-print, 7315 proteins were investigated specifically in chronic COVID-19 disease, relating to complement protein as key in the coagulation pathways; complement C1q subcomponent subunits A, B, and C (C1QA, C1QB, and C1QC) were mainly enriched in lungs and LNs [42]. Complement factors C3, C5, C7, and C9, in contrast, were commonly upregulated in LNs and aorta/vessel walls with downregulated SP-C in ATII cells [42]. Investigations indicate two other proteins, receptor for advanced glycation end-products (RAGE/AGER) and chloride intracellular channel (CLIC5), also associate with ATI cells, but that an SP-C related protein was downregulated specific to ATII cells, as above [42]. Interestingly, the authors noted a significant reduction in IL-12 production in LN that can affect DC maturation, as discussed below. Many cell cycle regulatory proteins were measured, such as a cyclin dependent kinase (CDK2), but also origin replication complex (ORC) and nucleoporins (NUC), which were observed to be upregulated in LNs. Furthermore, many proteins’ changes were noted as corresponding with tissue cellular changes within the glycocalyx. In a similar case control study looking at biomarkers in seropositive individuals (n = 400), significant changes also occurred to E-selectin (CD62) and cathepsin B, and that persistent symptoms may be associated with iron-sulfur cluster co-chaperone protein (HSCB), heat shock protein HSP 90-beta (HSP90AB1), amyloid-beta precursor protein (APP), phospholipase D Family Member 3 (PLD3), cystatin-C (CST3), and calprotectin (S100-A9) [43].

### 1.5. Pre-2022 Laboratory Research Context

Since 2015, research studies have clarified that SP could modulate host immune responses in lung inflammation and, therefore, may be a therapeutic target during increased dysregulation seen in chronic COVID-19 disease [44]. There is a discordance in the literature, and this may have been overlooked during the influenza 2009 H1N1 pandemic [44]. As numerous reports (n = 10) clarify, with SARS-CoV-2 infection, there is extensive alveolar damage with endothelial injury of cell membranes, vascular thrombosis, occlusion of alveolar capillaries, oedema with angiogenic vessel growth, and lymphocyte migration [44]. The resulting mechanisms controlling cytokine regulation occur between all leukocytes with resulting questions over immune cells and respective interleukins (IL), growth factors (GF), chemokines (CXC), and respective receptors or ligands (e.g., CXCR3 and/or CXCR4) that require further clarification below [45]. SARS-CoV-2 pathogenesis begins with disrupted membrane homeostasis with resulting syncytia formation, cell fusion, and multinucleate cells accompanied by immune system dysregulation [46,47,48]. This formation of syncytia could be initiated by transmembrane proteins (e.g., TMEM16) that regulate phospholipid rich cell membranes including phosphatidylserine (PS) [49,50,51]. Braga et al. utilized cell fusion inhibition assays (CFIA) and in situ measurement of viral RNA assays (n = 41) studies in affected individuals to clarify that SARS-CoV-2 infected individuals had fused cell syncytia dominant that contained napsin, which processes SP-B common to ATII cells [50]. They noted that, by regulating an ion channel dependent on calcium and one scramblase enzyme that regulates PS, the S protein clearly appears to activate transmembrane proteins (TMEM) at the cell membrane surface or within organelle membranes [49]. For example, one of these TMEM16 is part of a protein family consisting of calcium dependent ion channels responsible for PS regulation in a normally calcium- and arginine-rich layer [49]. Concurrently, it is now known that SARS-CoV-2 ORF3a may affect a calcium regulated ion channel, TMEM16F, regulated by PS, that can augment procoagulant activity through tenase and prothrombinase complexes, which are key regulators of the coagulation pathway [50,51,52]. Therefore, such localized changes infer routes of SARS-CoV-2 entry within the epithelial microenvironment. Indeed, the carbohydrate-rich glycocalyx layer covering mucosal epithelial cells also contains a mixture of mucin (MUC) glycoproteins, glycosaminoglycans, and other glycoproteins, which extend and surround cilia and normally function to clear larger bacteria. Expansive research recently revealed that cilia, microvilli, and mucus function remain key for SARS-CoV-2 adhesion and receptor mediated entry into epithelial cells, which appear to act as adhesives. MUC proteins are high-molecular-weight proteins that form mucus clusters. In COVID-19 disease, initially (n = 16) two types of mucins were extensively investigated, of which membrane-tethered MUC1 and the gel-forming MUC5AC appeared at significantly elevated levels. Therefore, normal pathogenic clearance via mucin proteins could be disrupted, facilitating SARS-CoV-2 entry to allow viral persistence [53,54,55,56]. Importantly, other research indicates that, in addition to ORF3a, other SARS-CoV-2 proteins include E and ORF8 that can act to assemble and form toxic ion channels [57,58].

### 1.6. Role of Toll-like Receptors (TLR) or TLR Induced IFN Dysregulation

In order to mount an anti-viral response, usually type I IFN is produced [59]. Current research contradicts this, as type I IFN production presents as beneficial and detrimental in COVID-19 disease; however, studies examining Middle Eastern respiratory syndrome (MERS) and respiratory syncytial virus (RSV) indicate that the timing of type I IFN production affects the cellular response [59,60]. Additional considerations are surface and cytosol pattern recognition receptors (PRRs) that initiate downstream signaling cascades utilizing NF-kB, type I IFN, and inflammasome pathways [43,44,45,46,61]. These include damage-associated molecular proteins (DAMP) that encompass a myriad of proteins surrounding and within nuclear and extracellular spaces that include ten conserved Toll-like receptors (TLRs), retinoic acid-inducible gene-I-(RIG-I)-like receptors, Nod-like receptors (NLRs), AIM2-like receptors, and intracellular DNA and RNA sensors, that lead to production of pro-inflammatory or anti-viral cytokines necessary for antigen specific adaptive responses [61,62]. For example, IL-1RA is a DAMP receptor, that, once released intracellularly, binds to and initiates IL-1α release, which is supported by case studies (n = 71) that showed this was the case in chronic COVID-19 disease concurrently with IL-10, which is largely immunosuppressive [63,64]. It is known that SARS-CoV-2 proteins are recognized by cellular sensors and, therefore, the roles of TLR3/4/7 are of interest in terms of which immune cells express these. TLR3 is more abundant in NK cells, whereas TLR4 is more common in Mϕ. Toll-like receptors (TLRs) transduce signals via MyD88 and TRIF. Most TLRs use MyD88 to trigger inflammatory cytokine production; TLR3 is the exception and signals exclusively through TRIF, whilst TLR4 is unique in that it can bind and signal through either MyD88 or TRIF to nuclear transcription factors. Previous *in vitro* studies indicate that TLR3/7 can be associated with IL-1α, IL-1β, IL-4, and IL-6 release [65]. Therefore, other studies investigated the nature of TLR7 as a risk factor in severe COVID-19 disease [66]. The role of TLRs in immune cell signaling is largely unclear, and will undoubtedly need further research, but is implicated in T cell signaling [67]. TLR4 presents on monocytes, Mϕ, and DCs, and in some non-immune cells, such as endothelial cells, and has a role in both LPS-induced Gram negative bacterial CD14 immune cell trafficking, and, interestingly, may regulate RORγt^+^ regulatory T cell responses in colitis [68,69,70]. Clinical trials regarding newer therapeutics affecting TLR are ongoing (NCT05089110, NCT04526977, and NCT05293236) in both COVID and HIV pathologies that will clarify this further (see Supplementary Materials).

The role of SP-A, as discussed above, is under research, and it is plausible that TLR4 expression has differential effects within select organ systems depending on activation, as seen in neonates, where TLR2/4 activation was shown to stimulate downstream extracellular-signal regulated kinase (ERK) and protein kinase B (AKT) with IL-6 pathways unchanged between children and adults [71]. Expression on both platelets and alveolar Mϕ could affect thrombotic and immune pathways simultaneously with reduced expression on ATII cells, and confirmation in animal studies, which have recently been shown to link TLR4 to intestinal cytokine mRNA expression [72,73,74]. TLR4 clearly has an influence on platelets through aggregation and P-selectin expression, and the formation of mixed aggregates between platelets and neutrophils, and in microbes with LPS triggers synthesis and/or secretion of von Willebrand factor (vWF), platelet factor 4 (CXCR4), and thromboxaneA2 (TXA2), alongside NETosis with CD11b upregulation and other adhesion molecules (Supplementary Data S1) [75,76].

Originally identified in 1957 by Isaacs and Lindemann, IFNs were found in secretions to inhibit viral and tumor growth. They are currently classified into three groups and individual subtypes: Type I, II, and III. Type I IFNs consist of IFN-α and IFN-β (also IFN-δ, IFN-ε, IFN-κ, IFN-τ, IFN-ω, and IFN-ζ) but also within Type II is IFN-γ, whilst type III IFNs encompasses IFN-λ [77]. With regard to SARS-CoV-2 sensitivity to IFN, early clinical case studies indicate SARS-CoV-2 sensitivity to IFN-α and IFN-β *in vitro*, however more recent tissue studies indicate that IFN-α and IFN-β response could paradoxically facilitate the viral propagation from the respiratory epithelium to the vasculature through direct endothelial cell infection [78,79]. Recently, type III IFN-λ has been investigated and is under clinical research following earlier studies (n = 257) that document reduced IFN-λ2 during chronic COVID-19 disease [80]. The cellular source of SARS-CoV-2 infection induced IFN production is largely unknown presently, as IFN receptors are located within B cells, monocytes, Mϕ, T lymphocytes, glial cells, neurons, and plasmacytoid dendritic cells (pDCs), among others [60,61]. Interestingly, epithelial response *in vitro* studies show that IFN-γ can promote SARS-CoV-2 infection in cell culture to enhance cell differentiation within enterocytes *in vitro* [81]. IFN-λ has been seen to be activated by bacteria, including *Staphylococcus aureus* [82]. It is of note that type II IFN and type III IFN can be secreted by NK and T cells, and few studies document whether type III IFN affects antibody class-switching. Therefore, as a key mediator of anti-viral responses within the respiratory tract, it is now being seen that IFNA2 and IFNG gene expression in the respiratory tract is accompanied by increases in IFNB1, and also with reduced early IFNA2, but that this IFN response seems to occur in sera rather than tissues [83,84,85].

## 2. Innate Immune Systems and SARS-CoV-2 Research

### 2.1. B Cell Development Dependency on T cell Activation

**Figure 2:**
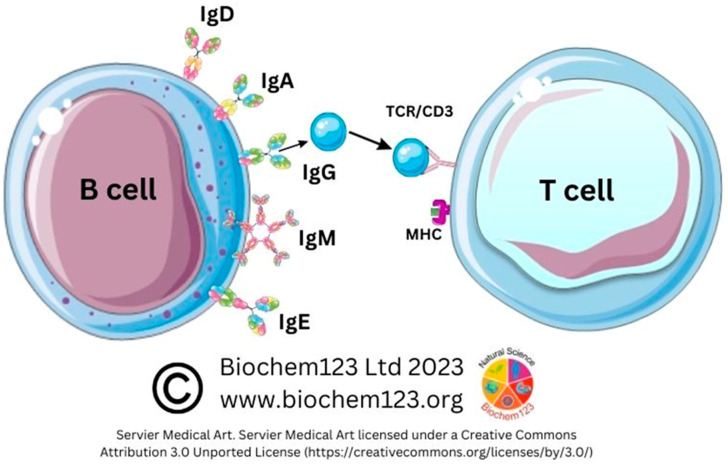
B cell and T cell interactions.

B lymphocytes represent 10% of white blood cells (leukocytes). Central to innate immune responses as pathogen sensors, these develop in germinal centers (GC) and then are distributed throughout the lymphatic system network by secretion of immunoglobulins (Ig), determining detection and neutralization of antigens through cellular development processes [86]. B cells respond to non-host antigens dependent on receptors that include antibodies shed from the cell surface (e.g., IgM, CD79a and CD79b) (see Figure 2).

**Figure 3:**
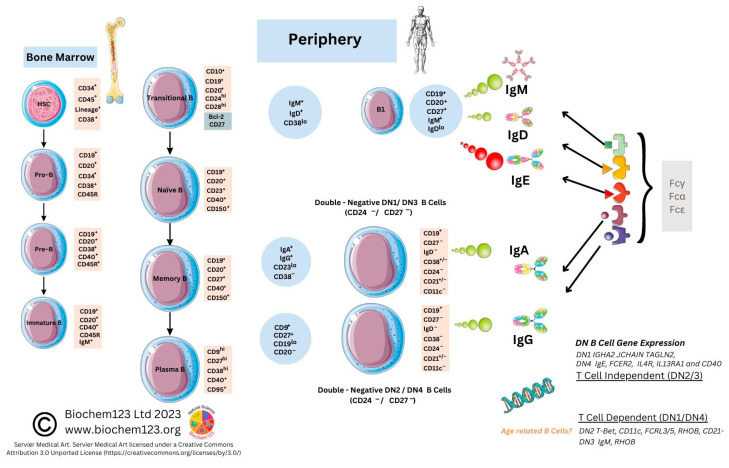
B cell phenotypes during maturation.

**B cell development from hematopoietic precursor cells (HPSC) occurs in stages from pro-B cells, pre-B cells, immature B cells, and growing into mature B cells in fetal liver and then in bone marrow**. B cell responses are defined by CD markers evolving into mature B cell subpopulations, such as B-1, B-2, and regulatory B cells [87,88]. Research on newer B cell subtypes defined by other phenotypic CD markers with single cell sequencing has occurred since 2017. Remarkably, B lymphocytes synthesize up to 10^11^ antibodies, or B cell receptors (BCR), within a host that undergo clonal selection and somatic hypermutation (SHM) leading to specificity of antigenic epitope protein recognition. BCR consists of a transmembrane section extending through cytoplasm with protein sequences that depend on co-activation or stimulation from other proteins to activate B cells. Other CD molecules define B cell development or lineage (e.g., CD19, CD21). These are relevant to cell-residing locations, developmental stages, maturation, and activation states. CD10 expression occurs on first-stage B cell lineage cells (e.g., pro-B, pre-B cell, and GC) and can change throughout maturation with others shown (see Figure 3) [89].

Moreover, CD27 exclusively resides within memory B plasma cells, whilst CD5 characterizes B-1 cells and DCs (see Figure 3). B cell receptor (BCR) complexes with other T cell markers (TCR) influencing maturation and antigen presentation result in pre-GC memory B cells (pre-GC MBCs) and short-lived plasma cells (SLPCs) that produce low affinity early antibodies. Other B cells reach the GC, where antibody affinity and selection can occur by clonal selection/SHM, modifying protein structure via class-switching recombination (CSR), resulting in long-lived plasma cells (LLPCs) and memory B cells (MBCs) with specific antibody isotypes, but also plasmablasts (PB) that produce Ig of the five main isotypes that occur as multimeric proteins (IgM, IgG, IgA, IgE, and IgD) in normal host-specific immune responses. These are indicated within these ranges in sera IgG: 80%, IgA:15%, IgM:5%, and IgD:0.2%, with trace amounts of IgE (see Table 1) [90].

### 2.2. Antibody Isotypes and Class-Switching

An antibody is a structural monomer unit of β-strands and complementary determining regions (CDRs), composed of two light chains and two heavy chains associating with each other through disulphide linkages secreted by B cells in response. However, a joining (J) chain is present within IgA and IgM, allowing formation of dimer or pentamer structures, which contrasts with other monomeric antibody isotypes. Light chain classes have constant and variable domains, and heavy chains possess five isotypes with individual roles associated in the immune system: IgG, IgD, and IgA (possess one variable and three constant chains) with IgE and IgM (one variable and four constant chains). The predominant immunoglobulin IgG (80%) is a tetrameric 150kDa quaternary structure globular protein with IgG occurring as four conserved subtypes (IgG1, IgG2, IgG3, and IgG4) and varying effector function [91]. Ig structures that encompass the BCR contain antigen binding F(ab) or constant F(c) structures, referring to the top polypeptide receptor or tail polypeptide structures spanning the phospholipid bilayer, and changes to either polypeptide conformation could affect either of the SARS-CoV-2/antibody transductions that effectively tether and affect downstream function of antibody molecules, either inside or outside the cytoplasmic membrane, in secretions or in sera. In short, an array of associated functions can result as individual isotypes known by Greek terminology, which have variable antibody isotypic receptors (e.g., FcγR denotes antibody isotype IgG receptor), resulting in an array of effector functions that can include phagocytosis, antibody-dependent cell-mediated cytotoxicity (ADCC), or complement fixation through other FcγR receptors (CD16, CD32, and CD64 and subtypes) [92,93]. IgG dominates during natural infection and vaccine responses in sera. IgA represents 15% of total Ig and co-exists in dimeric secretory form, with monomeric subunits adjoined by a 15kDa J chain in the upper respiratory tract. IgA1 and IgA2 secretion by B cells in infection usually occurs in ratios of 90% IgA1 to 10% IgA2 [94]. IgA is found in secretions including tears, saliva, sweat, colostrum, genitourinary tract, prostate, and respiratory epithelium, and in trace amounts in blood. Secretory IgA (sIgA) composition occurs in ratios within colonic secretions of IgA1-90%:IgA2-60% [95]. IgA production happens from plasma B cells actively being transported to mucosal surfaces, affecting activity via respective FcαRI (CD89) receptors present on neutrophils, basophils, eosinophils, monocytes, Mϕ, and DCs, modulating host protection from bacterial infection and sepsis. CD64 was characterized around 2015 as a sepsis biomarker that can occur within the gastrointestinal tract, prostate, and respiratory epithelium, and in trace amounts in blood [96,97,98]. IgA is key in mucous membrane pathogen clearance (e.g., *Haemophilus. influenza*) and ingested allergens such as peanuts. IgM to IgE class-switching occurs during allergy responses and hypersensitivity that can result in high IgE levels together with further mast cell degranulation, vasodilatory complications, and/or anaphylaxis. IgD class switching is important in B cell maturation in transition between autoreactive to antigen specific responses [99].

MHC class I/II receptor signaling complexes are found on many cells and antigen presenting cells (APCs) such as DCs, monocytes, and Mϕ, endothelial cells, as well as epithelial cells [100,101]. These facilitate extracellular peptides endocytosed and then digested within lysosomes, by presentation of peptides to the cell surface effector cells. The MHC class II complex is encoded by human leukocyte allele (HLA) genes, which are divided into subtypes including HLA-DR (also HLA-DM, HLA-DQ, and others). These are highly polymorphic and vary depending on genetic factors, thus the variable HLA gene complex affects presentation of SARS-CoV-2 antigens to effector cells with cytokine stimulation and antibody secretion [102]. The BCR protein complex non-covalently binds to other proteins and membrane-bound Ig receptor (mIg), Ig-alpha (CD79a), and Ig-beta (CD79b). This CD79a/CD79b complex transduces signals, giving linkage to mIg heterodimeric disulphide complexes that contain immune tyrosine-based activation motif (ITAM) sequences vital for B and T cell signal transduction. Immunoreceptor tyrosine-based activation marker (ITAM) protein sequences transmit activation signals from BCRs through tyrosine to the cell cytoplasm. Tyrosine phosphorylation by protein kinase cytoplasmic cellular signaling occurs with proteins. The inducible co-stimulator (ICOS) homodimeric protein promotes T/B cell signaling through CD40 and B7/CD40L. Such co-stimulatory interactions are vital for progressive adaptive immunity development and lymphocyte activation. Inefficient adaptive immunity can cause significant impairments in Ig isotype switching. Moreover, ICOS ligand B cell interaction with activated T cells is essential for the formation of GCs and optimal production and sustained release of other serum/sera antibodies (IgA, IgE, and IgG subtypes) to maintain effective host immunity. However, HLA genetic variations can be associated with differential risk factors in many disorders. Notably, MHC class I is expressed by nearly all nucleated cells [100,103].

### 2.3. Role of B Cell Markers in Current Research

CD19 has long been used as a B cell biomarker [104]. In recent years, this has expanded into characterization, as below, of B cells by receptors expressed at different maturation stages throughout B cell lineages, which include naïve B cells, unswitched memory B cells, switched memory B cells, and double-negative (DN) B cells, but also, alongside this, chemokine receptors responsible for systemic lymphatic direction. Some studies suggest there is no consensus on DN B cells; however, these recently characterized DN B cells cellular markers are clearer (see Figure 3 or Supplementary Data S2). Further DN B cell analysis of CD11c has refined these into subsets expressing CXCR5 hypothesized to emerge from naïve B cell activation outside of the GC [105]. Researchers expanded this recently to two other subtypes, DN3 and DN4 B cells (see Figure 3). Enrichment within certain DN B cell subsets has been discussed to play a key role in other comparatively well-characterized autoimmune diseases (multiple sclerosis (MS), systemic lupus erythematosus (SLE), myasthenia gravis (MG), and rheumatoid arthritis (RA) [105,106,107]. For example, CD27^+^IgD^+^ has been suggested to have impaired gene signaling in RA through VH3-23D to VH1-8, affecting production or, rather, reduced BCR diversity during selection [108]. Thus, recent subtypes have examined the DN CD11c B cell phenotype (n = 18) to show these in autoimmune pathologies compared to healthy controls (SLE, Sjøgren’s syndrome). Furthermore, B cells (CD19) expressing CD11c^+^ together with elevated levels of CD69, Ki-67, CD45RO, and CD45RA, as metabolic markers and B cell memory phenotype markers, along with a lack of DN cell markers CD21, could well escape normal immune cell regulation [109,110]. Therefore, depletion of B cell subsets has also been examined in age, referred to as age-associated B cells (ABCs), with regard to affecting the production of autoantibodies [111,112,113,114,115,116,117,118].

### 2.4. B Cell Antibody Responses during Respiratory Infections

IgG1 and IgG3 were initially associated with severe disease in older adults with COVID-19 (n = 123) disease and accompanying irregularities in neutralizing antibodies (nAbs), chemokines, and T cell responses, which is an anomaly as IgG3 was previously thought to provide enhanced pathogen response [91,119,120]. However, IgG deficiency has been observed to be associated with increased mortality risk in Chronic Obstructive Pulmonary Disease (COPD) patients (n = 489) in these ratios: 56% IgG1: 27%: IgG2: 24% IgG3: 31% IgG4 [120]. A previous study (n = 105) comparing serology of hCoV-229E, hCoV-OC43, hCoV-NL63, and hCoV-HKU1 elucidated that participants show in other hCoV antibody responses with regard to IgG that were 99%:100%:98%:91%, with IgA in nasal wash samples, detected in between 8% and 31% of participants [121]. Lower pathogenic hCoVs represent 15–30% of common cold respiratory tract infections in humans each year, also with seropositivity estimated at 90% in adults, indicating that T cell roles require further clarification [122]. A non-coronavirus antigen comparison would therefore be influenza virus that expresses haemagglutinin (HA) and neuraminidase (NA) proteins that present seasonally. In these cases, research indicates increases in serum IgM specific HA antibodies during the 2009 H1N1 pandemic in these ratios: IgM (86–94%), IgG (100%), and IgA (76 to 96%) [123,124].

### 2.5. B Cells and Antibody Responses to SARS-CoV-2 Infection

In late 2020, Plume et al. carried out a unique study (in brief, see Table 2) examining SARS-CoV-2 protein antigen fragments, analyzing these against antibody isotypes against most of the virus antigens, and they indicated that seroconversion at day 20 may cause issues, as 97.3% reacted against the chosen epitopes of SARS-CoV-2, but E protein was not investigated in this study [125,126,127].

The overall frequency of individual antibody responders within individuals (n = 103) in this study, which was conducted between April 2020 to January 2021, is indicated (see Table 2). Differential polyclonal antibody responses would normally occur as immune cells mature against different viral protein antigens. Therefore, frequency of individuals producing polyclonal antibodies against subtypes of SARS-CoV-2 S protein (S1, S2), M protein, and N protein antigens in pre-January 2021 samples was examined. This ranged with IgG dominant against RBD, S1, and M protein domains in 100% of their samples in moderate to severe disease (see Supplementary Data S3). Frequency of IgA responders was seen as 24.7–35.6%, reacting against whole S protein domain, RBD, S1, S2, M, and N protein domains, with the latter not predominant in severity. It is noteworthy that this study recognized limitations of IgE assay sensitivity at that juncture, and would be worthy of further investigation in comparable studies [126,127].

B cell memory and production of S-protein-specific Ig has been measured, and it can now be seen that a putative role for unknown B cell subtypes, such as DN2 B cells downregulating CXCR5, or other direction-specific receptors such as CD62L (L selectin) that are usually expressed, could affect the immune responses [125]. CD19^+^ CD24^+^ CD27^+^ CD38^+^ B cells indicate that antibody responses to SARS-CoV-2 S protein-specific B cells are increasing, although further details can be observed regarding the two other relevant transitional B cells (see Supplementary Data S2) [107,126].

In contrast with other studies, it was found that a 32 amino acid peptide (V551–L582) in the currently mapped RBD domain could be an immunodominant B cell epitope, equating to 58.7% of IgG samples tested [127]. However, E protein assays were performed in concurrent studies that did not appear, in historical analysis, to have similar viral neutralization properties pre–2020, indicating that prior exposure may not have occurred as epitopes differ in viral antigens. Initial plaque neutralization assays indicate that sufficient concentrations of nAbs may occur to S1, RBD, and potentially to N protein SARS-CoV-2 domains with predominantly IgG response. As above, naïve B cells expressing IgD^+^CD19^+^CD27^−^ (see Figure 3, Table 3, and Supplementary Data S2) can be sole predictors of antibody titrations compared to control groups with high significance (*p* = 0.009) [128].

Nonetheless, chronic COVID-19 disease patients presenting with DN (IgD^−^ CD27^−^) B cells are shown to experience worse disease severity and complications. The DN1 subset is noteworthy for holding potential for early activated memory cells, whereas the DN2 cells encompass antibody PB–secreting cells that have been primed beforehand; however, it remains uncertain what impact that each DN B cell subtype and mechanistic property has on disease resolution [129]. Interestingly, earlier in the pandemic, the nature of the SARS-CoV-2 S2 domain antibody response was indicative that it was comparatively immunogenic, stimulating both IgA and IgG. Statistically, eighty six percent (86%) of individuals were seen to have antibodies against this conserved S2 domain, with more concentrations of antibodies produced against S2 domain than the RBD domain [130].

SARS-CoV-2 can be considered novel by not eliciting a higher level of secretory IgA, such as both influenza and lower pathogenic hCoVs. In fact, chronic COVID-19 disease did stimulate elevated levels of five serum antibody types of IgM, IgG1, IgA1, IgG2, and IgG3 [131,132,133]. It was demonstrated in chronic COVID-19 disease that significantly higher IgG1, IgG2, and IgG3 occurred at day 3 alongside transient elevation in IgA1, disappearing by day 7; the impact of this is unclear, as are the cellular mechanisms that affect this, but research is ongoing. A concurrent study also confirmed that IgM–IgA1, IgM–IgG1, and IgM–IgG2 were enriched in acute SARS-CoV-2 infection, demonstrating an initial robust immune response [84]. Therefore, along these lines, IgA variability was investigated in sera to determine cellular phenotypes, including FACS analysis (n = 135) of PB B cells to show in acute infection that IgM and IgG were secreted between 10 and 15 days after infection, in these ratios: IgM: 10.5% (range 4.2–54.1), IgG: 27.9% (range 7.4–64.8). Therefore, B plasma cells (PBs) that produce IgA were further quantified by expression of proliferation markers and B cell markers Ki67^+^CD19^lo^CD27^hi^CD38^hi^ to produce IgA: 61.4% (range 18.1–87.6) occurring in these antibody subtypes, IgA1:66% (range 26.8–88.5), compared to IgA2: 31.6% (range 3.7–70.8), which are in line with above overall mucosal immune responses [134].

However, individuals with dominant IgA responses were seen to have an associated risk of mortality in severe COVID-19 disease, as below, that experienced dysregulated myelopoietic responses. High IgA to low IgG titrations can cause pathological consequences in the host, involving decreased pathogenic phagocytosis, increased cellular apoptosis, and increased NETosis, as reported in late–stage fatal COVID-19 disease. Individuals possessing a high IgG to IgA ratio experience increased inflammatory dampening through immune response, resulting in better prognoses and early–late–stage disease resolution. Limited data exists to reveal why SARS-CoV-2 induced COVID-19 disease exhibits such a novel antibody profile regarding IgG1/IgA1 responses. It is considered that alterations to the Ig structure can produce complications, either increasing infections or immune complex formation, and in other pathological diseases such as dengue antibody–dependent enhancement (ADE), IgG antibody structure was examined. These changes include glycosylation (glycan or carbohydrate adjoining hydroxyl or other functional groups) and fucosylation (transfer of a sugar fucose from a GDP–fucose to other proteins or glycans), and, therefore, this could affect leukocyte extravasation and selectin–mediated binding through cellular membranes, which is recognized as a potential factor in cancer therapeutics [135,136]. Therefore, studies during the pandemic (n = 33) examined this in chronic COVID-19 disease to confirm that IgG, against the SARS-CoV-2 RBD protein, could potentially affect Mϕ release of IL-1β, IL-6, IL-8, and TNF [137]. However, IgG3 and IgM are inferred to be responsible for 80% of the neutralization of SARS-CoV-2, with suggestions that IgG3 glycosylation affects SARS-CoV-2 binding specificity to the S protein [132,138].

As mentioned earlier, glycosylation can occur where an N–linked glycan forms within the IgG-Fc region. In accordance with examining the IgG subtypes, a recent study from Brazil examined the avidity of IgG (n = 47) to SARS-CoV-2 proteins to show an increase in IgG1 and IgG3 levels at day 8, and IgG4 concentration levels were less detectable during the study period. Mortality at 8–21 days showed higher anti–RBD IgG4 levels in comparison with the recovered, which contradicts other studies and is relatively unknown with regard to IgG4 pathology research [139]. Initial screens of N/S/E SARS-CoV-2 proteins in smaller cohorts (n = 320) indicated that anti-N IgG and anti-N IgA were produced in response to SARS-CoV-2, and IgG antibodies were produced to S1 and E proteins, but also that the anti–E protein antibodies evoked were not significantly higher, which is indicative of the current immunogens within clinical trials and those used in lateral flow testing [140]. Currently, *in vitro*, it has been determined that specific memory B cells are produced for up to 6 months, compared to S protein, with IgG measured at 66% and IgM at 100%, which would be consistent with either natural infection or vaccination [126].

In comparison to other pathologies, such as Ebola virus (EBOV), in survivors of the 2013–2016 outbreak, the roles of IgG1/IgA1 and IgA2 indicated a trend of polyfunctional IgG1 being more immunologically beneficial against each of the four EBOV proteins, being GP, secreted GP (sGP), nucleoprotein (NP), and matrix protein VP40, which offers biologic plausibility [141]. As above, such variabilities can further be explained, as it is thought that fucosylated IgG1 immune responses against SARS-CoV-2 antigens or E proteins vary, and variations at N297 could, in theory, affect the biological response through NK cells, monocytes, and Mϕ that each express FcγRIIIa (CD16a), as well [142]. Higher concentrations and/or production of IgG1 in severe COVID-19 disease was investigated in a laboratory study (n = 43) and found to be affected by reduction in Fc fucosylation (afucsosylation) with elevated IgG3, corresponding to the antibodies against SARS-CoV-2 RBD domain. Therefore, as antibody responses are polyclonal in nature, it suggests that within sera that contain polyclonal IgG3 and IgM, even though they are only present as 12% of overall Ig, these are responsible for 80% of virus neutralization against the N domain of SARS-CoV-2 [129,143,144]. The functional significance of this is in affecting lower affinity CD16a (FcγRIIIa) receptors, which are extensively researched in dengue fever disease, and that afucosylated antibodies affinity of IgG Fc to respective receptors could potentially influence concentrations of localized antibodies available, increasing immune complex formation when these are upregulated in disease [145]. This was later confirmed when researchers found a correlation between non–neutralizing, afucosylated antibody responses *in vitro* that stimulated monocyte production of pro–inflammatory cytokines IL-6, TNF, and IL-1β [146]. Therefore, this balance of FcγRIIIa (CD16a) and FcγRIII (CD32) remains crucial. These receptors play a role in host immunity via stimulation or inhibition of downstream signaling in cells that have differential affinities depending on the specific receptor expressed. As we outlined above, antibodies undergo selective subtype switching during maturation, and data is starting to emerge on the potential relevance of chemokine signaling to the antibody subtype produced. Interestingly, CCR6, which has one endogenous ligand, CCL20, is being considered in context with impairment studies and influence on both IgA and IgE responses with largely unknown mechanisms currently [147,148,149].

### 2.6. Role of B Cell Markers during SARS-CoV-2 Infection and Other Conditions

Antibody responses to SARS-CoV-2 immunogens can be attained in individuals on anti-CD20 therapy via the onset of B cell repopulation [150]. In the absence of B cells, a strong T cell response is nevertheless generated, which may help to protect against chronic SARS-CoV-2 induced COVID-19 disease in this high-risk population [150]. Therefore, it is essential to understand the nature of this response. Researchers in 2020 found, earlier in the pandemic, that within chronic COVID-19 disease (n = 52), affected individuals’ overall quantities of B cells did not significantly change. However, compared to healthy individuals, DN1 B cells significantly decrease with severity, with increases in DN2 B cells seen between moderate to chronic disease, but note a significant increase in patients during disease severity of DN3 cells, but with no concurrent changes in the DN4 B cells. Other B cell subsets were therefore investigated to discover a novel subset of B cells named “transitional B cells or T_R_” that may correlate with clinical outcome as measured by B cells expressing more CD24 than CD21 [129]. This was an interesting finding, because CD24 expression is known to affect cell migration, invasion, and proliferation, while expression or lack of CD21 is associated with B cell switching and memory with complement proteins. CD21 is also expressed on follicular dendritic cells and known to associate as a complex with complement proteins (C3dg, C3d, and inactive C3b) on the antigen surface, together with CD19/CD81 [115,129,151]. Interestingly, increased T_R_ cells did correlate with COVID-19 disease routinely used blood protein markers detected, including neutrophil/lymphocyte ratio, acute phase proteins, ferritin levels, D-dimer, and others. The exact nature of DN B cells requires further clarification, as subsets are associated with SLE [152]. As before, the B cell DN1 reduction/DN2 increase in chronic COVID-19 was accompanied by high levels of CD69 and CD89 in DN2 cells, alongside what appears to be DN2 selection of IgG, but also suggests that DN3 cells may produce VH4-34 IgG autoreactive antibodies, some of which could be protective [118]. There were initial indications that germline Ig variable heavy chain VH4-34 showed decreased SHM frequencies, which would affect B cell Ig maturation through the SHM process [153]. Unswitched memory B cells (CD27^+^ IgD^+^), historically, are part of normal and pathological immune responses with reduced overall IgM secreting B cells. For example, in RA, unswitched B cells are thought to occur due to gene recombination, contributing to antibody selection by VH3-23D to VH1-8 [108]. Interestingly, BCR repertoire of these cells was altered in RA, exhibiting some of the same markers as DN2 cells, such as CD11c, FcRL5, and transcription factor (T-bet) [154,155]. While antibodies generated by B cells are historically well-characterized, it is unclear why SARS-CoV-2 generates high antibody responses in chronic severity and not in acute infection. The timing of an antibody response is important in antibody-based therapeutics, as drug application influences patient outcomes [156,157]. Naïve B cells are activated with the assistance of follicular T (T_FH_) cells [158]. Therefore, this novel antibody expression caused by SARS-CoV-2-infection-induced chronic COVID-19 disease was found to be of three antibody classes and isotypes, including IgM, IgG1, IgA1, IgG2, and IgG3, which require further analysis. Recent analysis of SARS-CoV-2 S protein immunogens indicate Ig expression by spike-specific B cells at six months was produced in these ranges: IgG: 61.33–77.46%, with concurrent IgA: 3.04–7.37%, and IgM: 12.30–24.97%, to note significant reduction in IgG/IgA with significant increase in B cell-specific IgM at six months [159,160].

As discussed earlier, B cells develop in GCs and, through a small cohort study (n = 15), a role for circulating T_FH_ cells was elucidated to show S–protein–specific B cells develop through SHM. At five months, 66% of this cohort had B memory cells to vaccine immunogens, and this research suggested that there was a slight increase in nAb [160,161,162,163]. Concurrent with other studies, unsurprisingly, minor differences in memory–switched cells as the main population were represented (median: 59.92%) [163]. Their analysis examined SARS-CoV-2–specific B cell markers, CD27 and CD38, that were accompanied with a significant increase in CD27^hi^CD38^hi^ PBs. This occurred in recovered individuals compared to uninfected individuals at six months, with IgD^+^CD27^+^ and IgD^−^ CD27^+^ B cells that were significantly reduced in chronic SARS-CoV-2 infection [161,163]. The extra follicular response remains under investigation and Woodruff et al., in a cohort study, suggested that the DN2/DN1 B cell ratio may underpin some of the serology anomalies in severe COVID-19 disease with CXCR5 downregulated and CXCR3 upregulated. CXCR5 is a chemokine constitutively expressed on specifically B cells and T_FH_ cells responsible for directing B cells to GCs, while CXCR3 has multiple ligands, including CXCL8/9/10, but is preferentially expressed on T_H_1 cells and the majority of the T cell population, DCs, and memory B cells. Evidence is emerging that upregulation or changes in these and other chemokines (CXCR3, CXCR5, CCR7) in acute infection and downregulation in severity would be a further indicator relevant to maturation of DCs [162,163]. Statistical significance was apparent between antibody secreting cell (ASC) expansion with high levels of CD21^−^B cells independent of duration of infection [164,165].

B cells control antibody secretion, and reports indicate that IL-10 and IL-21are responsible for B cell class-switching to IgG1, IFN-γ class-switching to IgG2, and TGF-β switching from IgA1 to IgA2 responses [133]. Research shows that IgG1 and IgG3 (n = 123) correlate in chronic SARS-CoV-2 severity with a cytokine IL-1β response [119]. IgG2 is thought to be more relevant to bacterial responses to capsular polysaccharide antigens. Concurrent *in vitro* studies also indicate that SARS-CoV-2 IgA1 and IgG3 may have a protective neutralizing effect in SARS-CoV-2 infection [164,165]. Further research would be required to clarify this claim. Other studies (n = 82) confirm that in chronic SARS-CoV-2 infection, within seven days, serum antibody response is 60% IgA, 53.3% IgM, and 46.7% IgG, with IgG reaching 100% by day 2 [166,167]. Therefore, a single-cell transcriptomic study analysis of severe COVID-19 disease in detail highlights that DN1 cells express IgA2 genes, and can therefore potentially secrete IgA2, whilst DN3 B cells were seen to express IgM genes absent in DN2 cells, with DN4 cells possessing IgE genes and corresponding Fc receptor genes (see Figure 3) [118]. Interestingly, this raises the possibility that there are distinct T cell-independent and T cell-dependent pathways in B cells during COVID-19 disease, which is now suggested in other concurrent studies. IgE, therefore, can be produced by DN4 B cells, but serology from COVID-19 patients was not considered to be statistically relevant to this cellular subset [118,129,168,169]. IgE is known to cause mast cell degranulation through a higher affinity FcεRI receptor, and assay sensitivities require validation and development for this antibody normally seen in allergic responses with the IgG response predominant during infection. It is notable that the H2 receptor, present in gastric mucosa, brain, and mast cells, was targeted using an antagonist famotidine in trials, and was seen to have some effects in modulating SARS-CoV-2-infection-caused symptoms with synergism with macrophage T_H_2 cytokines [170,171,172].

## 3. Inflammatory Cells and Phagocytes

### 3.1. Neutrophil Introduction

Polymorphonuclear neutrophils (PMN) are granular and trilobed, being the most common circulating leukocyte, representing between 40% and 80% of leukocytes in normal adults. Neutrophil infiltration in respiratory tissues is characteristic of many inflammatory diseases [173]. Neutrophils are granular, acting against antigens by dispersing azurophilic cytoplasmic granules using the actions of proteolytic enzymes (e.g., myeloperoxidase, elastases, and proteinase–3) but also lactotransferrin, lysozyme, or reactive oxygen species (ROS), which are also anti–microbial, for clearing pathogens [174,175]. Pathogenic stimuli trigger cellular calcium release via endoplasmic reticulum (ER), resulting in activation of protein kinase C (PKC) and assembly of the NADPH oxidase complex generating ROS. Neutrophils form hematopoietic stem cells (HPSCs) in bone marrow and are short–lived, between 1 and 7 days, and traverse cell membranes by selectin–dependent capture and integrin mediated adhesion (see Supplementary Data S1), after which migration to tissues occur, and they survive for 1–2 days while circulating and clearing by phagocytosing Mϕ. Development of neutrophils occurs in bone marrow from progenitor neutrophils and can be broadly classified according to CD markers as CD81^+^CD43^+^CD15^+^CD63^+^CD66b^+^, which differentiate into immature neutrophils expressing CD11b^+^CD66b^+^CD101^+/−^CD10^−^CD16^+/−^ before maturing in the bone marrow to express CD11b^+^CD66b^+^CD101^+^CD10^+^CD16 [176]. CD16 is co-expressed on other cells including NK cells, monocytes, Mϕ, and certain T cells [176]. CD16 (FcγRIII) has subtypes including CD16a and CD16b (FcγRIIIa/FcγRIIIb), whilst CD11 and, specifically, CD11b are classified as more relevant to migration and lung inflammation [177,178,179]. It is notable that CXCR2 and CXCR4 appear to be key regulators within this cell subset implicated in lung fibrosis, but these also modulate cellular mitochondrial activity, neutrophil migration, and neutrophil homing utilizing adhesion receptors that include CD62L [180,181,182,183,184]. However, more recently, CD11b and CD18, which are ubiquitously expressed, are now thought to require CD47 in epithelial transmigration [185,186]. Interestingly, Alberca et al. examined a novel cell subtype, defined as myeloid derived suppressor (MDSC) cells, within laboratory case studies: CD33^+^CD11b^+^HLA–DR^−^CD14^−^CD66b^+^ and CD33^+^CD11b^+^HLA–DR^−^CD14^+^CD66b^−^cells. In peripheral blood markers of chronic COVID-19 disease, this was seen to correlate possible M–MDSC and polymorphonuclear P–MDSC, which have been linked to chronic inflammation [186]. These MDSC–defined cells have been described initially in cancer, HCV, and HIV to affect T cell proliferation. Recent clarification on putative phenotypes emerging as M–MDSC (CD11b^lo^D14^+^CD15^−^HLA–DR^−^) and P–MDSC as CD11b^lo^CD14^−^CD15^+^ HLA–DR^−^ have been suggested to affect T_REGS_ as below through TGF–β affecting the overall balance of both T_REGS_ and self–tolerant DCs [187].

### 3.2. Neutrophil Cellular Markers after Host SARS-CoV-2 Infection during COVID-19 Disease

During chronic COVID-19 disease, it is believed that neutrophils form neutrophil extracellular traps (NETs), as we discussed above, where the cell signaling within is disrupted, causing active degranulation or “NETosis/neutrophil apoptosis” [188]. The exact mechanisms of NETosis contribution remain unknown [189]. Therefore, recent case studies (n = 64) focused on identifying neutrophils cellular markers further. During COVID-19 disease, some appear to suppress stimulation of IFN-γ production with unknown cell subsets that stimulate T cell proliferation but fail to activate T cells [190]. Several authors suggest host driven immune response is causal in lack of production of type I and III interferons in conjunction with elevated chemokines, and IL-6 is a causal factor in coronavirus pathology [191,192]. Whilst IL-6 could be the predominant cytokine regulator of NETosis, other protein markers are clearer, those being extracellular DNA (ecDNA), neutrophil elastase (NE) activity, or myeloperoxidase-DNA (MPO-DNA), and these correlate with disease severity, measured in neutrophils by markers CD33^lo^CD16^+^CD11b^+^ [193]. Researchers recently found (n = 155) that NE, histone-DNA, MPO-DNA, and free double stranded DNA (dsDNA) were increased with concurrent DNase reduction and exacerbation of neutrophil stimulation occurring via IL-8, CXCR2, and DAMPs with impaired degradation of NETs via DNase 1 and DNase 11L3, which are suggested to act as regulators of neutrophil DNA metabolism [194]. Recent abstracts also imply a correlation between these and either membrane bound or soluble CD13 [195]. A comprehensive neutrophil analysis (n = 384) utilizing single cell analysis classified six cell states defined by inflammatory gene signature (IGS) to indicate that concordant IgA1:IgG1 ratios are elevated in coronavirus disease mortality, with IgG indicating antibody-dependent neutrophil phagocytosis and IgA2 inducing apoptosis [133]. Interestingly, neutrophils expressed significantly increased levels during maturation of CD32 (FcγRII), CD16b (FcγRIIIb), and CD89 (FcαR), the main Ig receptors which have respective ligands on B cells, above. No known studies currently exist connecting type III IFN with this isotype switching. A similar investigation into IgA2 (n = 97) confirmed that anti-SARS-CoV-2 IgA2 in severe COVID-19 disease correlated with ecDNA [131]. Syncytia formation and NETosis are likely in the formation of immune complexes as an imbalance due to or caused by coagulopathy and immunothrombosis [131,193]. Endothelial cells in both animal and human studies revealed endothelial cells could be directly infected; however, studies show colocalization with CD31 within a disrupted inflamed endothelial layer, as clearly seen by upregulation of many adhesion molecules (e.g., P-selectin) and chemotactic factor release of CXCL10 alongside IL-6 (see Supplementary Data S4) [196,197]. Additional factors involved in platelet coagulation are vWF, with elevated P-selectin and E-selectin upregulation, observed in chronic COVID-19 disease patients, that are all involved with endothelial dysfunction [76,198]. Exploring this further, Kuchroo et al. performed a single cell analysis study (n = 168) of infected SARS-CoV-2 patients that differentiated between neutrophil and monocyte populations with monocyte markers (CD16^hi^CD66b/CD14^−^CD16^hi^HLA–DR^lo^) to find T helper 17 (T_H_17) cell response generated IFN-γ and granzyme B [199]. In this key finding, CD14^−^CD16^hi^ monocytes were enriched in severe infection, and it was confirmed that HLA-DR upregulation correlated with severity. In 2020, it was shown in chronic COVID-19 disease patients that IL-2, IL-4, IL-6, IL-10, TNF-α, and IFN-γ with C-reactive protein (CRP) correlated with IL-10 [34]. IL-1 is a key cytokine involved with neutrophil activation, which shares homology and similar functions with aforementioned TLR families [64,200]. IL-1α and IL-1β have been implicated in COVID-19 disease in other studies. Therefore, the exact mechanisms of the regulatory enzyme glycosyltransferase, α-1,6-fucosyltransferase (FUT8) were examined, only to find little correlation with disease prognosis, but if was found that receptor expression was upregulated within other myeloid monocyte compartments that express CD16a (FcγRIIIa), also including classical (CD14^hi/+^, CD16^−−^) and intermediate (CD14^hi/+^, CD16^lo/+^), and non-classical (CD14^−−/lo^, CD16^+^) markers that also include CD11c DCs that are also HLA-DR^+^ myeloid cells [146,201,202,203,204]. Clinical trials remain ongoing to further clarify cytokines IL-1 β, IL-6, IL-8, TNF-α, TGF-β, IFN-γ, IL-17, IL-21, IL-22, IL-23, and IL-10, and ROS production in COVID pneumonia, with results awaiting (Supplementary Data) (NCT04930757, NCT04434157, and NCT05520918). As above, neutrophil proteins disrupted during NETosis naturally will affect extracellular ions and, specifically, calcium homeostasis required by other cytoskeletal proteins with intracellular enzymes, not necessarily degraded, that include MPO, histones, and other proteases within this cytokine and immune cell environment [205].

### 3.3. Monocyte Cellular Development

**Figure 4:**
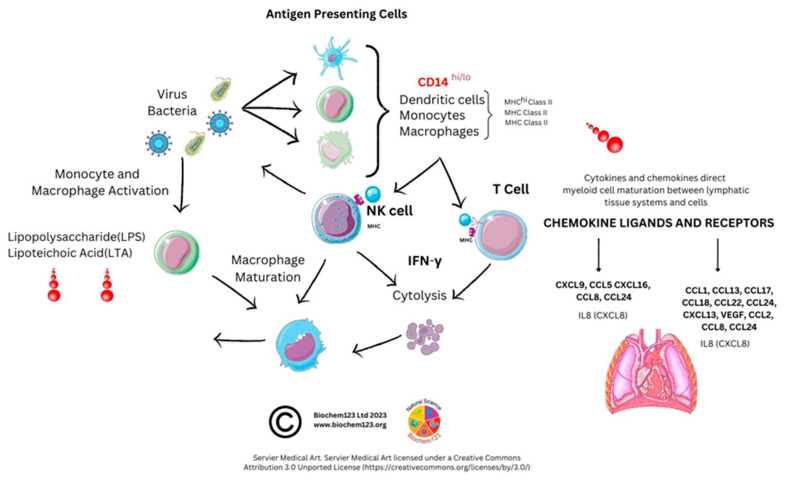
Antigen presenting cell roles in SARS-CoV-2 infection.

Since the advent of FACS and discovery of monocytes by Ehrlich and Metchnikoff, currently identified monocyte subsets are broadly defined by classical (CD14^hi/+^, CD16^−^), intermediate (CD14^hi/+^, CD16^lo/+^), and non-classical (CD14^−/lo^, CD16^+^) markers [203,206,207]. Monocytes represent around 10% of the leukocyte population, and they are short-lived (1–2 days) while circulating in blood, bone marrow, and spleen (see Figure 4).

In keeping with other cells, they also undergo apoptosis to form a cluster of APCs, including DC and Mϕ, developing during inflammatory insult. They phagocytose viral antigens alongside antigen presentation (using MHC class II proteins). By synthesizing and secreting cytokines, monocytes are therefore central to immune responses. Monocytes also have reactive receptors to extracellular cytokines and chemokines to become inflammatory DCs or Mϕ, with their development affected through TLRs and PAMP stimulation. Monocyte expression of MHC class II molecules (HLA) affects antigen presentation to T cells. In response to stimuli, monocytes have a differential response, and *in vitro* stimulation found classical monocytes CD14^++^ and CD16^−^ responded to IFN-γ, GM-CSF, and IL-4, and that intermediate monocytes could change the MHC II (HLA-DM) presentation that associates with TCR, but also that GM-CSF can modify MHC II HLA-DR on CD14^+^CD16^−^monocytes, which are a key dominant inflammatory subset with the hypotheses that non-classical CD14^+^ and CD16^++^ monocytes could be involved in patrolling endothelial vessels and be affected by IFN-γ changes, as below. Importantly, IL-10 can downregulate MHC class II molecules on monocytes, but specifically HLA-DR on all monocytes and HLA-DM on CD14^+^ and CD16^+^ [208]. Alternative classical monocytes can secrete proinflammatory cytokines such as IL-6, IL-8, and chemokines CCL2, CCL3, and CCL5 [209,210]. Throughout monocyte development, progressive changes in activation and upregulation of CD16 expression can occur with antigen presentation complexes usually accompanied by cytokine secretion of TNF-α, IL-1β, and IL-6 upon TLR stimulation. Subsequent COVID-19 laboratory analyses, provided below, were implemented to clarify context surrounding these unique cells.

### 3.4. Monocyte Cellular Markers during Host SARS-CoV-2 Infection

**Figure 5:**
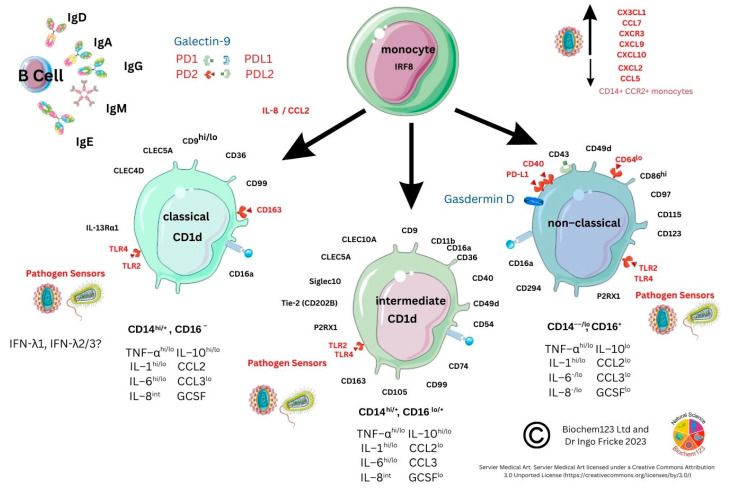
Monocyte cell phenotypes.

In COVID-19 disease, it was intimated that classical CD14^++^CD16^−−^ monocytes were a source of upregulated chemokine CCR2, along with a neutrophil chemoattractant IL-8 (CXCL8) and TNF-α with upregulated gene expression and synthesis of IL-1β and IL-18 with fewer confirmed CD14^+^CD16^++^ monocytes. Moreover, downregulation of HLA-DR in severe patients (n = 12) was seen to affect overall viral antigen presentation [201,211,212]. These cellular populations are further characterized by CD195 (CCR5), as well as TNF-α receptors CD120a/CD120b (TNFR1/2). Both of these receptors were found in blood serum and up-regulated along with ADAM17, known to affect L-selectin (CD62) shedding, with ADAM17, a TNF-α convertase, also upregulated in inflammatory bowel disease (IBD) [213,214]. Other soluble immune cell shedding markers were measured in sera (sCD14 and sCD163), and, although unrelated to disease severity, they correlated with standard blood sera proteins (acute phase protein, ferritin, LDH, CRP, and procalcitonin) [215]. In addition, through CCR5 inhibition studies, during prolonged SARS-CoV-2 infection and disease, this demonstrated that changes to CD14/CD16 subsets occurs, affecting pro-inflammatory cytokines alongside CD4^+^/CD8^+^ T cell reduction. These researchers showed that IL-2, IL-4, CCL3, IL-6, IL-10, IFN-γ, and VEGF were elevated and, moreover, T_REG_ cells decreased with concurrent GM-CSF reduction, affecting monocyte development [216]. FACS analysis was used for NK cell analysis and in differentiating CD14^hi/+^, CD16^−−^monocytes by CD16 marker to find occurrence via inflammasome activation (NLRP3) evidenced by caspase-1 activity in severe COVID-19 disease. This concurred with dysregulation of mitochondrial superoxide and lipid peroxidation markers of oxidative stress. These findings were later confirmed during gasdermin D cleavage studies [217]. Gasdermin D (GSDMD) is known as a pore forming protein significantly found to be activated by SARS-CoV-2 infection of neutrophils, as measured by caspase 1/3 activation as a potential NETosis and pyroptosis stimulator [218,219]. In addition, 6% of SARS-CoV-2 infected monocytes were found to have other pyroptotic markers when measuring GSDMD, IL-1β, IL-1RA, IL-18, and LDH, as well as three key chemokines: CCL7, CXCL9, and CXCL10 (see Figure 5) [220].

*In vitro* studies showed this could be causal in IL-1β secretion by SARS-CoV-2-exposed monocytes [221]. Specifically, the numbers of circulating classical monocytes (CD14^hi/+^, CD16^−^) were enriched with downregulation of CCR2 and HLA-DR, but the numbers of intermediate (CD14^hi/+^, CD16^lo/+^) and non-classical (CD14^−/lo^, CD16^+^) monocytes increased [222]. Alternative transcriptomic analysis confirmed that intermediate CD14^hi/+^CD16^lo/+^ possessed a temporal interferon-stimulated gene signature (ISG) in acute SARS-CoV-2 infection (IRF7, IFI44L, IFIT1, and IFIT3). Analysis also, remarkably, illustrated that IL-8 (CXCL8) and IL-1β together with CCL3 were substantially upregulated without induction of pro-inflammatory cytokine genes such as TNF, IL-6, IL-1, CCL3, CCL4, or CXCL2 in the cells that possessed reduced HLA-DR expression and reduced antigen presentation capability [223]. On the other hand, more recently, in a SARS-CoV-2 case control study (n = 37), it has been clarified that there was an initial increase in classical monocytes (CD14^hi/+^, CD16^−−^) with a decrease in intermediate (CD14^hi/+^, CD16^lo/+^) and a gradual normalization of non-classical monocytes (CD14^−/lo^, CD16^+^) 6–7 months after follow-up, with changes to other cell subtypes below [203,204,207,224].

### 3.5. Macrophages Metabolism and Function

**Figure 6:**
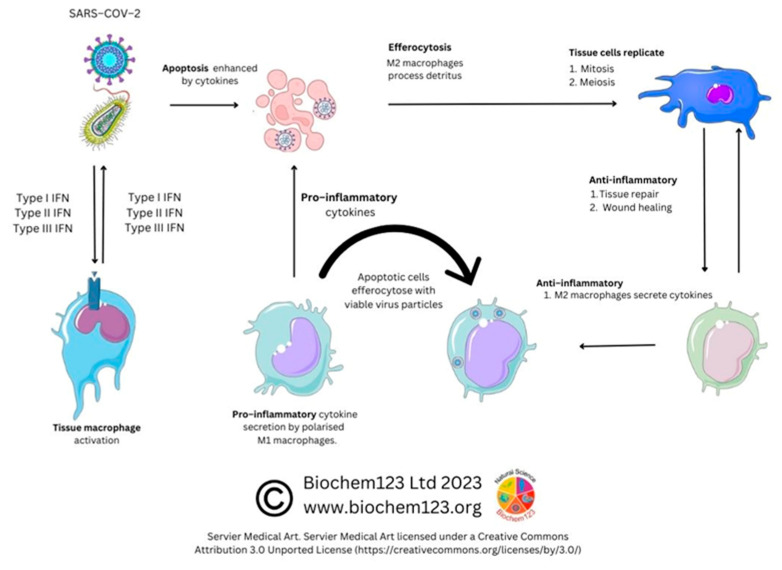
Macrophage process and role in infection.

In 1950–1970, macrophage (Mϕ) metabolic cycles were closely examined in what was then known as the Warburg effect, where Mϕ in tumors were seen to change metabolic profiles. Indeed, recent research shows that activation of Mϕ or DCs with a range of stimuli (LPS, TLR3 ligand poly (I:C), type I IFN) induces a metabolic switch. Metabolic profiles thus change from oxidative phosphorylation (OXPHOS) to glycolysis with resultant reduction in the TCA cycle, while lactate production drives Mϕ metabolism and fluxes upwardly through the pentose phosphate pathway [225]. Mϕ are the most abundant immune cell type within the lung, classified as alveolar ϕ (AMϕ) or interstitial (iMϕ). Macrophages (Mϕ) originate from blood monocytes that migrate between vascular tissues with morphology recognizing TLRs, pathogen-associated molecular patterns (PAMP), and pathogenic antigens. Conclusions are difficult to draw with reference to Mϕ interactions with B/T cells, as explained below (see Figure 6).

### 3.6. Macrophage Classification

**Figure 7:**
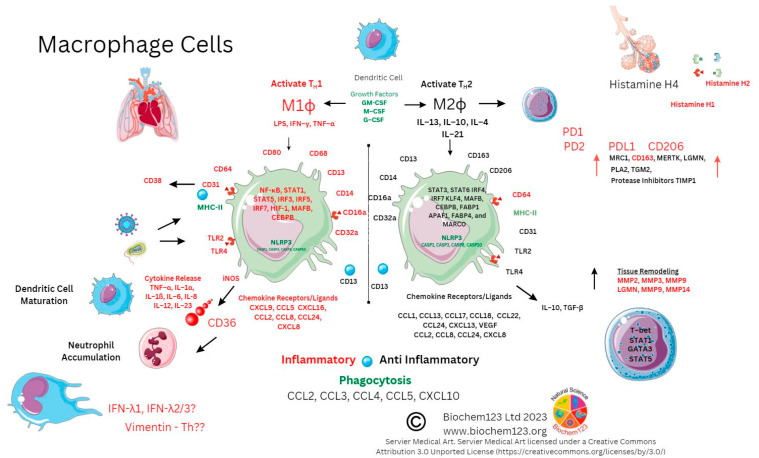
Macrophage phenotypes during polarization.

Less data has defined interstitial macrophages (iMϕ), in comparison to alveolar macrophages (AMϕ) being well-defined as regulators of lung pulmonary immune responses. AMϕ and iMϕ are both tissue-resident phagocytic cells that also include brain microglia, liver Kupffer cells, and others. Therefore, AMϕ are distinct in their ability to induce and inhibit inflammatory responses on exposure to pathogens, and change cell surface markers utilizing complement opsonization receptors and other pattern recognition molecules, as above, that facilitate phagocytosis of either cell debris or pathogens [226]. Mϕ characterization subsequently loosely differentiates between varying inflammatory phenotypes, commonly referred to as M0 (non-activated), M1 (pro-inflammatory), and M2 (anti-inflammatory) by polarization and cytokine secreted but are currently not defined by CD nomenclature [227,228]. As M-CSF and GM-CSF induce differentiation, it was suggested that Mϕ are subdivided into M1ϕ secreting cytokines IL1-β, IL-6, IL-12, and TNF-α, with M2-like secreting TGF-β, IL-10, IL-4, and IL-13 (see Figure 7) [229].

Individual classifications within M2ϕ revolved around T_H_2-like cell activation or suppression. Up until around 2017, classifications of Mϕ seemed to diverge from M2ϕ into 3 further subtypes (M2aϕ, M2bϕ, and M2cϕ), reflecting differential polarization of cellular states changing with technological understanding of gene transcription factors, leading to either cytokine secretion or by the actions of cytokines released by other cells (See above Figure 7), as these are the predominant circulating phagocyte. Macrophages (M1ϕ or M2ϕ) at that juncture were considered to be anti-tumor or tumor associated (TAM), undergoing changing maturation and polarization [229,230,231].

### 3.7. Macrophage Metabolism Role during Polarization and SARS-CoV-2 Infection

**Figure 8:**
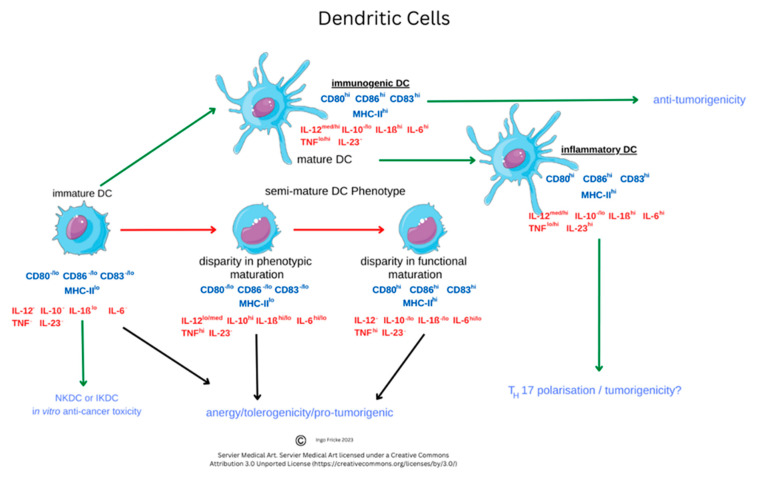
Functional diversity of dendritic cells in maturation.

**Figure 9:**
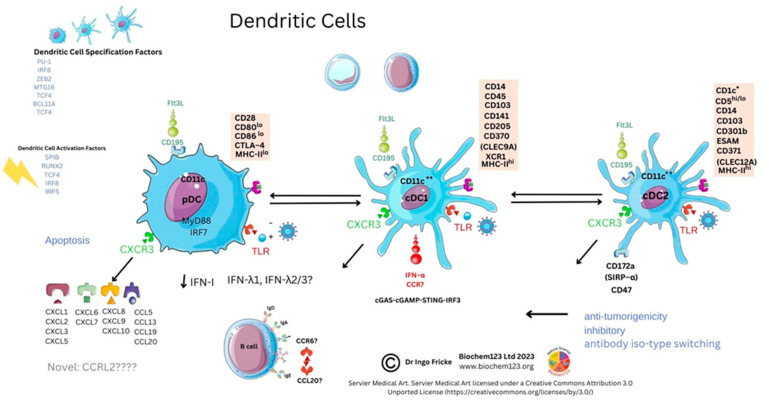
Dendritic cell phenotypes.

**Figure 10:**
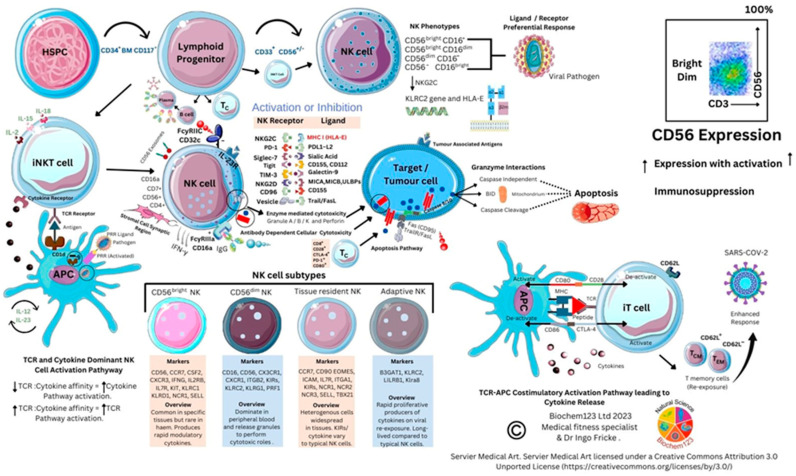
Natural killer cell phenotype diversity and maturation.

**Figure 11:**
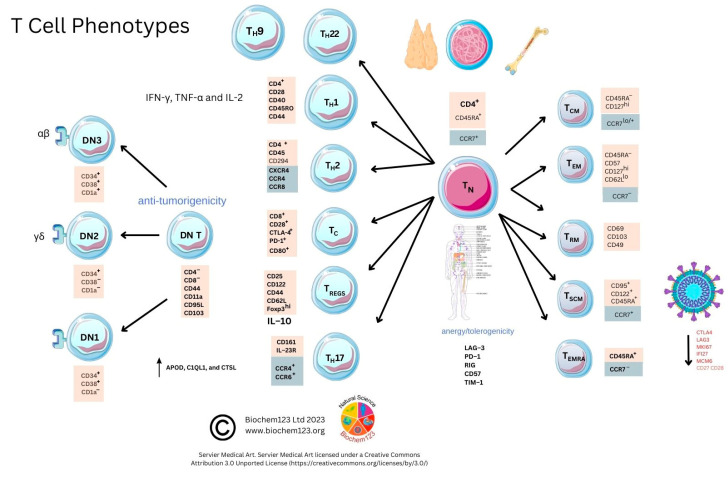
T-Cell phenotype diversity and developmental cellular markers.

**Figure 12:**
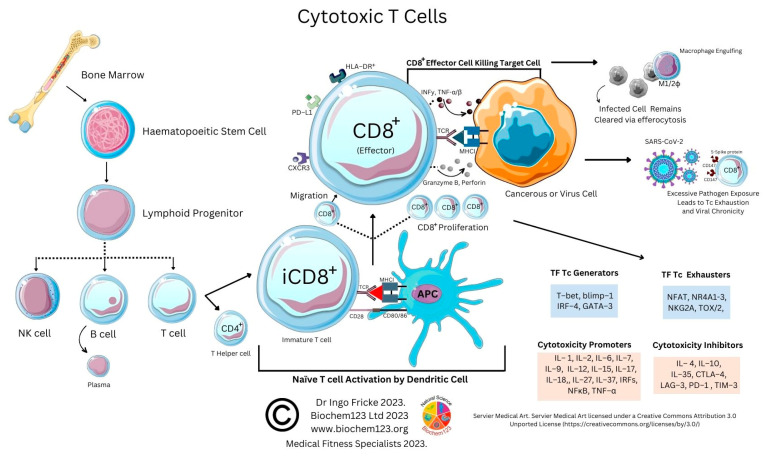
T cell phenotype diversity and developmental cellular markers.

Macrophage polarization is the process where Mϕ evolve through maturation and adopt different functional and secretory programs in response to signals from the microenvironment in which they are located at a given point in time. This dual innate and adaptive capacity relates to multiple roles in all organisms as effector cells involved at the center of most biological processes. More specifically, they are involved in the elimination of cellular debris, pathogens, embryonic development, and tissue repair utilizing an array of immune system cells that include B lymphocytes, DCs, T_H_1, T_H_2, NK cells, and others, below (see Figure 1, Figure 2, Figure 3, Figure 4, Figure 5, Figure 6, Figure 7, Figure 8, Figure 9, Figure 10, Figure 11 and Figure 12) [232]. It is noteworthy that IFN-γ is thought to polarize M1ϕ, causing up-regulation of inflammatory cytokines upon viral infection whilst inhibiting growth and enhancing apoptosis of lung cells *in vitro* [233]. Dysregulation of AMϕ polarity therefore needs to be considered in context with other *in vitro* or *in vivo* respiratory research where both fibrosis and inflammation can occur (e.g., silicosis) [234]. M1ϕ and M2ϕ, along with gene protein markers within bronchoalveolar lavage fluid (BALF), are one way of ascertaining polarity state alterations associated with disease. Cellular staining methods such as hematoxylin/eosin and trichrome staining on lung tissue can alternatively be utilized. M1ϕ/M2ϕ phenotypes appear to undergo differential phenotype changes affecting T cells and the resulting Ig class switching, as well as differential antigen presentation alongside chemokine and cytokine release within both respiratory and mucosal compartments, are affected by the factors below. M1ϕ can produce nitric oxide synthase (iNOS), which uses L-arginine to produce nitric oxide (NO) while M2ϕ utilizes arginase 1 (ARG1), which hydrolyses L-arginine to L-ornithine for collagen synthesis. Therefore, during infection and/or phagocytosis of Mϕ, changes in extracellular metabolites could occur, affecting polarization and changing the oxidative phosphorylation balance dependent on amino acid consumption. Furthermore, it is possible that M1ϕ metabolizing extracellular arginine into NO and L-citrulline with increased glycolysis, fatty acid synthesis, and ATP metabolism could change the levels of metabolites. In comparison, M2ϕ showing enhanced OXPHOS and glutamine metabolism therefore represents a metabolic cellular shift that could occur similarly [235,236,237]. Research is comparatively unclear on whether M2ϕ activation is glycolysis dependent. Therefore, metabolism in COVID-19-affected individuals is essentially relevant to immune cell function where patients’ decreased tryptophan was seen with elevations in L-kynurenine, which usually increases with age [238]. Tryptophan is an essential amino acid regulated by enzymes indoleamine 2,3-dioxygenase-1 (IDO-1) or indoleamine 2,3–dioxygenase–2 (IDO-2), eventually leading to producing kynurenine. Researchers clarified IDO-2 in a case control cohort study (n = 21) with a similar pathology to confirm that both IDO-1 and IDO-2 occurred in abundance within and outside AT1, AT2 cells, interstitial, and endothelial cells, with IDO-2 being largely localized to the lungs rather than tissues. Selected immune cells (Mϕ, DCs, and neutrophils) migrate within the respiratory compartment in SARS-CoV-2-infection-induced COVID-19 disease [238,239]. Therefore, this apparent confirmation that IDO was expressed in disease, along with the limited availability of other research, suggests that known selected M2ϕ markers IL-10/CXCR4 may increase, while T cell homing receptors CCR7 and IL-12A (IL-12p35) have been known to decrease in other fibrotic conditions [240]. M1ϕ and M2ϕ phenotypes are clearer with exposure to other *in vivo* bacterial and viral agents [241,242]. Fibrosis occurs around vascular compartments and within endothelial layers, and is understandably linked to COVID-19 disease and long-term sequelae where tissue stiffens, with concurrent decreased oxygenation and lung dysfunction. For example, Galectin-3, as a carbohydrate-binding protein, is produced in the lungs by AMϕ and epithelial cells. M2ϕ secretion of TGF-β or IL-10 can therefore either stimulate secretion of tissue-modeling proteins or regulate T_REG_ cells in acute lung injury. TGF-β is considered to act synergistically with -AMϕ in secretion of retinal dehydrogenase (RALDH), an enzyme that catalyzes the retinal to retinoic acid conversion within the cell, which is critical to the transcription factor retinoic acid-related orphan receptor gamma t (RORγt) [235,243,244]. Recent literature suggests that M2ϕ is dependent on higher energy production [235,236,244]. Therefore, as outlined above, the only other relevant immune system cells, being mast cells, basophils, and eosinophils, are discussed elsewhere, but were investigated in 2021.

This unique study in context examined allergic responses to document *in vivo* rhinitis allergy, appearing to clarify a clear pathway for T_H_2 and Mϕ cells in histamine induction through H1 histamine receptors, causing rhinitis, compared to H4 receptors being receptive to eosinophilic accumulation. These are known pathways that could be affected by the release of IgE, exacerbating allergic responses or mast cell degranulation that can occur in seasonal co-infections or allergies. As in SARS-CoV-2, much research is relatively unknown with regard to IgE [171]. In comparison, in other studies, similar AMϕ surface pattern recognition markers that were observed to exist on AMϕ were CD206^hi^CD86^hi^. The respective M1ϕ/M2ϕ-like phenotypes (CD206^lo^CD86^hi^ and CD206^hi^CD86^lo^) represented less than 1% of the entire AMϕ cell population. CD206^hi^CD86^hi^ subtypes possessed greater CD163 M2ϕ marker levels, in comparison to M1ϕ and M2ϕ. Analytical experiments also found CD80 and CD64 M1ϕ markers, and also found that HLA-DR activation markers were highly expressed by CD206^hi^CD86^hi^ Mϕ compared to other AMϕ subsets (See Figure 7) [228,245]. Of note, phagocytes have differential properties in tissue compartments, such as RA, but cells expressing CD14 can also include tissue resident DCs, as below [246]. Recent studies seem to confirm that both M0ϕ/M1ϕ express CD68/CD80, with CD163 as a highly specific mannose receptor expressed by M2ϕ. *In vitro* studies highlight that these were expanded in SARS-CoV-2 samples, which is indicative of expansion of M1ϕ and M2ϕ phenotypes, but no difference was found in CD206^+^HLA-DR^−^ or CD206^−^HLA-DR^+^ Mϕ as AMϕ/IMϕ populations in other infections [233]. CD68 appears to be a heavily glycosylated protein that is postulated to have ligands within oxidized low-density lipoproteins (LDL), PS, and apoptotic cells in atherogenesis, while the CD80 natural ligands are CD28, as outlined below, affecting T cell checkpoint proteins [247]. Other reports confirm that, in severe cases, variable expression of Mϕ markers, above, occurred with cells that did not express the usual T cell markers, those being CD3^−^, CD4^−^, or the non-glycosylated marker CD20^−^ [248].

### 3.8. Macrophage Phenotypes, Cytokines and Chemokines during SARS-CoV-2 Infection

SARS-CoV-2-infected Mϕ *in vitro* were seen to colocalize at endothelial cell membranes, expressing CD31 (PECAM-1) alongside endothelial cell endosomes and also displaying activation markers to exosomes expressing mRNA for IL-1β, caspase 1, and NLRP3 from infected individuals [249]. It is notable that complement opsonization receptors include CR1/CR2, but also exosomes CR3 and CR4 (β2 integrin), as well as CD11b/CD18 (αMβ2) expressed on neutrophils that can bind iC3b being an efficient phagocyte receptor, although many integrin subunits are also upregulated (see Supplementary Data S1).

Therefore HLA-DR (encoded on chromosome 6p21.31), was found to present S protein antigens and combinational peptide units of S1/S2/RBD, so this represented a key finding that antigen presentation was occurring [233]. Interestingly, both Mϕ and MDSC express CD68 and CD163, which were investigated in 2018 in the context of thrombocytopenia (ITP) to try and clarify MDSCs phenotypes further. Initial indications that chemokine receptors and ligands directed leukocyte migration were evident with CCL2/CCL3 and eotaxin. It was also indicated that IL-1β may expand both these cell types within ITP patients prior [250]. Further single-cell sequencing of SARS-CoV-2 within other inflammatory disorders (RA/CD/UC) clarified that, in BALF samples during COVID-19 disease, preferential expression occurs of CXCL10, CXCL9, CCL2, CCL3, and IL-1β (also GBP1, STAT1 gene proteins). These were also induced by IFN-γ and TNF-α, therefore clarifying that M1ϕ are pro-inflammatory in COVID-19 disease. However, Mϕ subpopulations are further characterized by HLA-DR, CD195 (CCR5), and TNFR1/TNFR2 expression, which is also higher on intermediate monocytes, followed by classical and then non-classical monocytes as well as Mϕ [251]. A recent preprint suggests that, within acute SARS-CoV-2 infection, monocytes change IGS from innate immune functions as CD14^+^ monocytes develop into pro-thrombotic, showing differential upregulation of MHC II alongside MHC I downregulation (HLA-DR/HLA-ABC), with accompanying gene signatures downregulated that would affect IFN production (e.g., IFNA1, IFNA2), but also TLR7 and AIM2, affecting increased expression of pathways involved in hemostasis and immunothrombosis [207]. In contrast, TNFR2 is expressed at high levels on non-classical monocytes, followed by intermediate, and then the lowest expression was of classical monocytes [252]. Enlarged monocytes with M2ϕ characteristics also secrete IL-6, IL-10, and TNF-α, and express surface receptors CD11b^+^, CD14^+^, CD16^+^, CD68^+^, CD80^+^, CD163^+^, and CD206^+^/CD14^hi/+^. CD14^hi^CD16^−^ Mϕ were observed to display inflammasome activation, as evidenced by caspase-1/ASC-speck formation in severe COVID-19 disease when compared to mild or healthy controls [221]. It is established that M2ϕ are T_H_2-like and can produce allergic cytokines, which are related to tissue remodeling and pathology that includes IL-4/IL-13. However, histamine H1 Mϕ receptor and eosinophil H4 also share this role [171,253]. CD68 and CD163 increase in severity alongside CD163 and T_REGS_. It is possible that M2ϕ, together with suppressor T_REGS_, promote this immunosuppressive environment. However, it is of note that other studies found that both M1ϕ/M2ϕ phenotypes could markedly upregulate CD38^+^ CD23^+^ in disease, which can prime DCs and naïve T cells [254]. Moreover, it was clarified by gene protein analysis that differential M1ϕ or M2ϕ polarization could be induced in vitro with M1ϕ expressing IL-6, TLR4, CXCL9, CXCL10, and CXCL11, while M2ϕ expressed CD206, CCL17, and CCL22 (with gene markers STAT6, IRF4) [233,255]. This was an interesting finding, as TLR4 is historically activated by bacterial antigens, while CCL17 and CCL22 seem to be relevant as DC and Mϕ chemokines. Therefore, on Mϕ, it appears that, aside from M1ϕ secreting IL-1β, IL-8 and IL-18, additional chemokines are expressed, such as CXCL16 together with CCL2, while anti-inflammatory M2ϕ expresses transglutaminase 2 (TGM2), apolipoprotein E (APOE), α2-macroglobulin (A2M), CCL13, and CCL26. Interestingly, a role for a triggering receptor expressed on myeloid cells 2 (TREM2) protein in potential toxicity of M1ϕ cells that hold affinity for the CXCR3 receptor seems clearer than before. TREM2 has been shown to be expressed on newly differentiated Mϕ, acting as sensor and activator of T cell responses in SARS-CoV-2 infection. Recent notable preprints confirm a role of TREM2 and iMϕ in orchestrating respiratory inflammation [256,257,258].

CD163 and CD206 are markers for the identification of Mϕ and CD68 and CD163, potentially more so for M2ϕ. Compared with CD68, CD163 is more selective to Mϕ, so CD163 can be used as a highly specific marker for M2ϕ. During SARS-CoV-2 infection, a reduction in overall leukocyte count and function can occur and has been observed by single cell analysis. However, other studies indicate depletion of CD16^+^ monocytes in peripheral blood, with CD14^+^ monocytes detecting no substantial induction of pro-inflammatory cytokine gene protein production, such as TNF-α, IL-6, IL-1β, CCL3, CCL4, or CXCL2 in these cells [222]. Furthermore, concurrent *in vivo* research shows that E protein can suppress inflammasome priming and NLRP3 inflammasome activation with reduced expression of pro-IL-1β, levels of IL-1β, and IL-18 in BALF, with Mϕ infiltration in the lung detected through NLRP3/caspase-1/IL-1β signaling [259]. As discussed, there are two types of Mϕ, with M1ϕ considered pro-inflammatory and stimulated by either IFN-γ and/or lipopolysaccharide. On the other hand, M2ϕ is considered anti-inflammatory, induced by extracellular IL-4. Intracellularly, M1ϕ has a lower endosomal pH that favors membrane fusion, allowing viral RNA entry through endosomes into the cytoplasm, where viral replication packaging and release occurs. In contrast, M2ϕ has a higher endosomal pH but a lower lysosomal pH, thus delivering the virus to lysosomes for degradation. This was indicated, for example, in hACE2 transgenic mice exhibiting distinct uptake, amplification, and release of SARS-CoV-2 by M1ϕ and M2ϕ, with preferential increased viral loads within M1ϕ and reduced N protein expression within M2ϕ [227]. Therefore, prior intracellular inflammasome suppression or activation can be seen in sequencing studies to confirm intracellular activation of caspases (CASP3, CASP 8, CASP 10) that are upregulated in iM1ϕs or iM0ϕ and undergo apoptosis within 48 h during infection [233]. Single cell analysis confirmed this with dexamethasone, remdesivir, and anti-IFNAR2 therapeutic observations *in vivo*; all of them have effects on respective gene regulation by intracellular activation sensors in this subset of cells. Furthermore, monocyte and M1ϕ/M2ϕ accumulation has been observed utilizing immunohistochemical analysis *in vitro*, showing that M1ϕ/M2ϕ can be directly infected by SARS-CoV-2, but it is currently thought that infection is permissive and occurs without active viral replication in these cells [260].

Therapeutics trialed and utilized before and during COVID-19 disease (e.g., methylprednisolone and dexamethasone) naturally can have non-specific anti-inflammatory and specific functions to decrease inflammation in different pathologies affected by multiple cellular pathways [226]. Therefore, some of the COVID-19-disease-related pathologies, such as lung fibrosis that is associated with alveolar injury, oedema, hypoxia, and inflammation, would, in effect, be reduced while the immune system responds; however, these are subject to local pharmacovigilance and monitoring studies [261,262,263,264].

## 4. Dendritic Cells

### 4.1. Dendritic Cell Overview

Dendritic cells were formally identified in 1873 (Langerhans cells), and by Steinman and Cohn in 1973 *in vivo* in the spleen, based on unique morphology, with a finite lifespan of days, distinguishing them from Mϕ, and they are replenished by hematopoiesis from precursor HPSCs [265]. Initially found to be potent stimulators of the mixed lymphocyte reaction, this elucidated their role as central to antigen presentation by expression of high levels of MHC class II molecules and integrin CD11c [202]. Therefore, in combination with migration ability between non-lymphoid and lymphoid organs, they hold a key with superior capacity to affect T cell development and function. DCs can be defined by migration to secondary lymphoid tissues and priming T_N_ (naïve) cells in combination with Mϕ in primary lymphoid organs. In 1994, a key development came in research describing *in vitro* cell culture methods for developing DC-like cells from monocytes using GM-CSF and IL-4 [266,267]. Compared to other APCs, such as Mϕ and B cells, these are considered the most efficient APC priming T cells via both MHC class I/II molecules and delivering antigens to CD4^+^ and CD8^+^ T cells. DCs develop from naïve to mature from a combined monocyte/DC pool that were thought to be CD103^+^ DCs that could process influenza virus in LN networks by cross-presentation, and were potent stimulators of CD8^+^ T cells [268]. DCs are formed from a heterogeneous population of bone-marrow-produced cells classified as: plasmacytoid DC (pDCs), type 1 conventional DCs (cDC1), type 2 (cDC2), myeloid DCs (mDCs), and Langerhans cells, but also monocytic DCs (MoDC) with CD14 subtypes above that evolve from hematopoietic stem progenitor cells (HPSC) (see Table 4).

Consequentially, in 2017, scRNA sequencing confirmed a further subtype cDC3 [269]. However, naïve/mature DCs can be distinguished by chemokine gradients and a checkpoint marker CD83^+^/^−^ together with CCR5^+^ (CD195) or CCR7^+^ determining migration towards or away from lymphoid organs and/or peripheral tissues. Distinguished by circular morphology, DCs undergo activation and/or development through PAMPs, PRR, and TLRs recognition of self and/or pathogenic antigens, causing signal transduction and concordant increase in intracellular calcium alongside nuclear factor (NF-κB), with progressive upregulation of CD80, CD86, and CD83, and developing into traditional cellular tree-like dendrites with several maturation markers as detailed below (see Figure 8) [270].

### 4.2. Role of Dendritic Cells and Cellular Markers in Disease

DCs and thymic epithelial cells involvement in tolerance in lymphoid organs and periphery is through continuous exposure to self-antigens with selection and elimination of high affinity self-antigenic recognizing reactive thymocytes (see Figure 9).

Steady state DCs undergo endocytosis and phagocytose both antigens and apoptotic cells while maturing in conjunction with stimuli, directing T cells towards deletion and anergy. Lineage polarization occurs, and also presentation of antigens to B and T cell phenotypes, which include T_N,_ T_H_, T_C,_ T_REG_ and T_H_17. It is notable that CD83 can be shed (sCD83), and is conserved amongst species but can be expressed on activated immune cells and others (B/T cells, monocytes, microglia, and neutrophils). However, CD83 has concordant effects associated with IL-12. Through stimulation of TCR with TGF-β, CD83 can colocalize with CD25, which could potentially stabilize T_REG_ CD4^+^Foxp3^+^CD25^+^ development [271,272]. However, viral infection as seen with herpes simplex (HSV), HTLV, and HIV can have variable modulatory effects on CD83, with deletion in T cells shown to enhance T_H_1 and T_H_17 responses, while, in contrast, deletion of CD83 from B cells stimulated by bacterial pathogens tends towards IgE/T_H_2-like responses. Therefore, CD83 can be considered a checkpoint marker which is currently under investigation in autoimmune conditions [272]. Individual DCs expressing high levels of MHC class II can encounter up to 5000 T cells per hour, perpetuating balance between autoreactive, tolerogenic, and inflammatory phenotypes through opsonizing self-antigens for cross-presentation throughout the lymphoid system [273]. These DCs are broadly characterized as CD3^−^ CD5^hi/lo^CD14^+/−^ CD19^−^. Recently, further CD5 characterization on DCs *in vivo* has occurred that regulates both CD4^+^ and CD8^+^ T cells [274]. This dichotomous role in tolerogenic responses extends to cancer responses due to duplicity of tissue-expressed tumor antigens (TAA), whereby tumor-infiltrating DCs (TIDCs) can suppress steady state DCs and prime T_H_ cells [275]. Malignant cells inherent genetic instability creates novel intra-host antigens, thereby dysregulating homeostatic immune responses. Therefore, presentation of TAA in the context of DCs can also occur within individual types within tumors. pDCs represent around 0.1–0.5% of nucleated cells dependent on one cytokine receptor ligand Flt3L that is a growth factor for stimulation and development of multipotent and/or lymphoid progenitor cells that include cDCs or pDCs [276].

These DCs express TLR7 and TLR9, which recognize ssRNA and unmethylated CpG motif-containing DNA motifs, respectively. Characteristic IFN responses include massive immediate secretion of type I-IFN, which commences within 1–3 h (including IFN-β and IFN-α subtypes) but also type III IFN through cGAS/STING and RIG-I pathways of intracellular sensing to both DNA and RNA viruses. Indeed, IFN secretion was examined with *in vitro* Epstein–Barr Virus (EBV) studies that illustrate secretion of the range of type I IFN (α1, α2, and others) specifically from pDCs that undergo maturation into mDCs. Within EBOV and HIV-1 infection, the IFN-induced receptor SIGLEC-1 has been implicated as upregulated in other diseases, either dependent or independent of TLR4, which curiously recognizes sialylated gangliosides on viral envelopes within the locality of CD209 (DC-SIGN) receptors, with maturity of DCs clearly known to affect infection avidity within these cells [277,278]. Studies have identified the connection between peptide presentation of MHC class II molecules expressed on endothelial cells, but also in autoimmune diseases [279]. In different autoimmune diseases, DCs carry certain HLA class II molecules with the risk of autoimmune disease, preferring the presentation of special antigens that will be recognized by the self-reactive TCR [280,281]. For example, in the case of type 1 diabetes (DM1), the presence of specific amino acids in the connector groove of the HLA-DQ8 supports the connection of insulin-induced peptides [282]. Similarly, in the case of RA, HLA-DR4 molecules play a role in the activation of specific CD4^+^ T cells [283,284,285]. It has been shown through cDC and pDC phenomena and function that in underlying genetic disorders or a chronic inflammatory environment, there is an association with development of various autoimmune diseases, such as RA, systemic lupus erythematosus (SLE), multiple sclerosis (MS), or diabetes mellitus type 1 (DM1) [286,287]. DCs can trigger or suppress reactive T cell responses. Their effects depend on DC subset, maturity degree, signals derived from the local microenvironment, and interaction with other cell types. This also includes abnormal intrinsic tolDC function, only now being examined in coeliac disease, where increases in CD11c, CD103, CD207, and indoleamine 2,3-dioxygenase (IDO) are increasingly likely to play a role in celiac disease [288].

The importance of DCs in immunological homeostasis also lies in the ability to regulate cytokines TGF-β, IL-10, IL-2, and IFN-γ. DC hyperactivation can lead to DM1 and is also caused by environmental triggers, such as inflammatory cytokines induced by bacteria or in response to viral infection, which can also be caused by excessive IFN production [289]. Furthermore, it is becoming increasingly apparent that certain subtypes of cDCs are more important than others. For example, cDC1 cells are not considered to be abundant within the tumor microenvironment (TME), but are becoming evident in NK driven CD8^+^ T-cell-driven responses as potential improvement on existing therapeutics that could affect squamous cell carcinomas of the head and neck, as well as melanomas of other types [290]. Tendency towards cDC1 immunogenic maturation and cross-presentation is maintained by type I-IFN and CCR5 expression by cDC1 that directs T_C_/NK responses through TLR3. However, antigen-specific CD8^+^ T cells cannot directly eliminate malignant cells, but this cross-presentation allows DCs to deliver exhausted or self-antigens on MHC class I/II molecules to CD8^+^ T cells. For example, cDC1 is primarily associated with cross-presentation of tumor antigens to CD8^+^ T cells and T_H_1 phenotype polarization of CD4^+^ T cells [291]. Both types of cDC1 and cDC2 can produce IL-12 after TLR stimulation. Tumor infiltrating cDC1s (TIDC) also produce different chemokines (CXCL9/10), facilitating migration of CD8^+^ T cells into the TME [292]. Therefore, elevated levels of TIDC can inversely correlate with tumor grade and stage with a robust prognostic value in multiple cancers, including non-small-cell lung carcinoma (NSCLC), melanoma, renal cell carcinoma, breast cancer, ovarian, and colorectal carcinoma [293]. IL-12 and IL-23 are two key cytokines in DC development (see Figure 9) [293]. It has also been illustrated that changes in one cell surface marker (e.g., CD5) can lead to over expression of IL-12 and differential T cell priming [274]. This can be seen within the cDC2 subtype, where CD5^hi/lo^ expression appears to clarify that IRF4 in CD5^hi^ cDC2 lymphocyte cells within the LNs acts as an inducer of maturation to produce higher levels of IL-10, IL-22, and IL-4 from both T_N_ and T_REGS_, with CD5^lo^ cDC2s inducing less IFN-γ [294]. On the other hand, early chemokine research within other viral diseases, such as HSV, extensively investigated routes of activation independent of T_N_ cells, utilizing upregulation of CCL2, CCL3, CCL4, CCL5, and CXCL10 on pDCs as inducing T and NK cell migration [295]. Therefore, identifying the individual chemokine changes produced by each cell type remain of value in determining contributions towards lymphatic cell migration to specific inflamed tissues (see Supplementary Data S4). More recent articles suggest that IRF8 derives different DC lineage maturity with lymphoids linked by CD123 in pDCs and B/T cell activation (BTLA) and myeloid lineages (DC3 and monocytes) expressing less of this transcription factor with CD33 (Siglec-1) and SIRP-α with AXL^+^SIGLEC6^+^ distinct to DC2s [296,297]. However, localized expression of TNF within epithelial cell environments has long been considered as a marker of inflammatory conditions that can affect not only T cell differentiation, but is also expressed within a variety of epithelial cell layers [298].

### 4.3. Dendritic Cell Role in Immune Responses to host SARS-CoV-2 Infection

DCs are difficult to culture and analyze, however, they can be obtained from monocyte lineage precursors or cultured DCs. In COVID-19 disease, it has been observed that monocyte/DC lineages were expressing complement genes (CD16^+^C1QA/B/C^+^) with CD34^+^ HSPCs (that can develop into monocytes, DCs or Mϕ) significantly upregulating MKI67 and TOP2A (proliferation and topoisomerase gene markers). The aforementioned genes increased with disease severity with complement C1QA/B/C^+^CD16^+^ expression on CD16^+^ monocytes, the first step in the serum complement cascade [299]. Longer term DC responses to acute SARS-CoV-2 infection were observed to show reduced DC numbers in laboratory research (n = 109), indicative that pDC expressing CD86 and either PD-L1^+/−^ that with CCR7 upregulation showed reduced IFN-α function through TLR9, which was found to be significant compared to healthy controls [300,301]. Multiplexed immunofluorescence assessed the impact of SARS-CoV-2 on the population of professional antigen-presenting cells (APC) in the lungs of COVID-19 autopsy cases in different stages of diffuse alveolar damage (DAD); this indicated that SARS-CoV-2 may impair the maturation of DCs. An increased count of myeloid dendritic cells (mDCs) was seen in later stages and accumulation of immature mDCs, which are unable to home to LNs adequately through cellular membrane receptor changes, resulting in inadequate T cell responses [302]. Therefore, DC-SIGN (CD209) expressed on both Mϕ and DCs and in other pathologies was investigated as a regulator in high mannose-expressing pathogens (e.g., *M. tuberculosis* and HIV) [303]. CD209 was found to be decreased in lungs but increased in blood of COVID-19 DCs, with cDC2 decreased in both lung and blood in disease but increased in lungs with severity. Furthermore, pDCs showed a notable increase in CD209 in BALF with accompanying depletion in sera in mild, but not severe, cases, and therefore chemokine homing ligands would reflect the migration changes of pDC from blood to lung in mild and severe COVID-19 [303]. Therefore, duly within the lungs, CCL2, CCL4, and CCL8 were seen to increase in severity accompanied by downregulation of HLA-DQA2 in severe cases, of which two of these share the CCR5 receptor, as above [295]. Immature DC subsets were seen to undergo changes to maturation markers (see Figure 9) with CD83 decreasing in tissues alongside MHC II downregulation therefore indicative of anergic or tolerogenic DC phenotypes. Furthermore, in a recent case control study, Hopkins et al. found (n = 37) that while both pDC and cDC1 subsequently normalized, cDC2 and cDC3 remained elevated at 6 months alongside lesser studied MDSC elevation, with changes occurring to HLA-DR, CD86, and PD-L1 in all cellular subsets [224]. It is notable that cDC3 cells and others were only defined around 2017 by scRNA analysis and, consequently, refer to these as expressing CD1c with monocyte markers CD14 and CD11b, but they are currently considered CD5^−^CD163^+/−^CD14^+/−^ [269,304]. In comparison with bacterial infection, cDC2/cDC3 subtypes with non-apoptotic genes AXL and CLU were examined. It was proposed that during inflammation, cDC2 progressively downregulated MHC class II, but also that a cDC2 and cDC3 phenotype shift occurs with gene protein expression of coagulation factors (C1QB, C5AR1, THBS1, THBD, ALOX5AP), production of arachidonate 5-lipoxygenase activating protein leukotrienes (ALOX5AP), and those coding for factors involved in vasodilation (ADM), but also S100A8/A9/A12 and ADAM9 [305]. Furthermore, as a key cytokine relevant to DCs, it has been shown that within COVID-19 disease, IL-12 was significantly increased in acute, but not severe disease, but also in hypertensive individuals as well [306,307]. With regard to the cellular source of three key cytokines in inflammation within the respiratory system, recent research has indicated that neither myeloid derived nor DCs produce IL-6, TNF-α, or IFN-α [308]. Therefore, systems immunology approaches measured all relevant factors that could have resulting effects on DC maturation (n = 61) to all the T cell maturation lineages that could affect T_H_1 cytokines (IL-12, IFN-γ, CXCL10, CD25 (IL-2Ra)) and T_H_2 cytokines (IL-4, IL-10, IL-13) together with T_H_17 (IL-17), together with IL-1. Interestingly, they identified an upregulated serum neutrophil marker CD177 with high significance increasing in concentration from symptom onset to admission with severity independent of other associated risk factors, although described in sepsis, that is a known neutrophil activation marker [309]. All APCs express MHC class I and/or class II transmembrane proteins, which present variable length intracellularly processed peptide antigens in conjunction with both BCR/TCR or respective localized receptors through processing, which remain key human leukocyte antigens (HLA) that play roles in tissue typing. Genetics risk factors have been observed with certain HLA alleles in many conditions due to genetic polymorphism, of which HLA-DRB1*04:01 in severity remains a risk factor in SARS-CoV-2 infection [310].

## 5. Natural Killer Cells

### 5.1. Natural Killer Cell Overview

NK cells are cytotoxic lymphocytes that provide innate immune defense against viral infections and cancer, representing around 10% of circulating leukocytes. NK cells were originally classified in 1989 and later phenotypically into CD56^bright^ CD16^−^, forming most NK cells, and a further three including CD56^bright^ CD16^dim^, CD56^dim^ CD16^−^, CD56^dim^CD16^bright^, and CD56^−^ CD16^bright^ with the latter CD56^−^ CD16^bright^ NK cells expanded in other pathologies such as human immunodeficiency virus and cancer [311,312,313]. Initially, NK cells were described as sensitive to IL-2-dependent activation and proliferation characterized by CD56/CD16 markers, with 2 cytokines TNF-α and IFN-γ preferentially produced in the CD56^bright^ NK cell subtypes [311]. Most NK cells (90%) express CD56^dim^ CD16^bright^ markers. CD56^bright^ NK cells were found to express CD2, CD11c, CD44, CD49, CD54, and CD62L, with CD56^dim^ NK cell expression of CD11 distinguishing respective differences with CD56^bright^ subset preferentially migrating between lymphoid system organs/nodes or inflammatory sites [312]. Research clarified the role of CD16a (FcγRIIIa) as a potent cytotoxicity receptor on NK cells, and CD16a (FcγRIIIa) has affinity for IgG1 and IgG3. Therefore, antibody recognition via opsonization of IgG targets can occur via CD16a (FcγRIIIa) recognition of IgG-opsonized targets. NK cells trigger downstream potent cytotoxic mechanism via ADCC and independent mechanisms leading to cell death utilizing lytic granules, dependent or independent of perforin and granzyme B release, to lyse the cell target, but also IL-2 can stimulate NK cell proliferation and expansion [314,315,316]. However, NK cell receptors also possess independent ligands that activate target killing, independent of lytic granules, such as Fas ligand and TRAIL, which may activate NK cell cytotoxicity. Furthermore, recent clarification of the enzyme caspase-8 intertwining the three major cell death pathways, including apoptosis, pyroptosis, and necroptosis, is suggested, given recent research that indicates its role in both T cells and NK cells [317]. Much is unknown about NK cell antigen presentation, however, recent *in vitro* studies report that increased HLA-DR expression is associated with NK cell proliferation activity, and IFN-γ production with higher expression of CD86 and NKG2D [318,319]. Therefore, NK cells are currently still identified as lymphocytes expressing CD56, although other researchers indicated that NK cell CD7 and CD4 can be expressed, separating these from other APCs such as DCs and monocytes. CD16a (FcγRIIIa) is notable in contribution to ADCC (see Figure 10) [320].

### 5.2. Natural Killer Cell and the Immune Response to Host SARS-CoV-2 Infection

During SARS-CoV-2 infection, and because NK cells are present at higher levels in lung BALF analyses, it was shown that CD56^bright^ and CD56^dim^ NK cells displayed an activated effector phenotype, with CD56^bright^ NK cells exhibiting higher levels of granzyme B, CD25, HLA-DR, and Ksp37 that are all necessary for cell mediated lysis, as well as antigen presentation [321]. Furthermore, this was corroborated by other studies demonstrating that NK cells, as measured by CD56^+^CD57^+^ and PD-1, were present at higher levels in naïve individuals [299,322,323]. Higher levels of soluble CD25 at the same time were found in BALF analyses (n = 280), indicating this is driven by delayed clearance of SARS-CoV-2 infection and expansion of CD25^+^PD-1^+^ CD8^+^ T cells [324]. Very recently, although not currently peer reviewed, through immune cell profiling PD-1 was confirmed to be preferentially expressed with downregulation of HLA-DR (n = 215) [325]. Interestingly, other single cell studies indicate that NK cell function is impaired and CD56^dim^ cells possess similar gene signatures (IFI6, ISG15, IFI44L, and FCGR3A) found in both sera and BALF to express NKG2A, PD-1, and CD39 in inflamed NK cells. However, interestingly, a subset was found expressing an inhibitory receptor (TIGIT) that could produce more IFN-γ with IFN-α2 of longer duration, thereby indicating other cell checkpoints could be more important in affecting this within both NK and T cells relevant to cell regulatory mechanisms [326]. Current research undertaken in clinical trials, from 2020, includes a phase one (NCT04634370) clinical trial for infusions of activated NK cells. In addition, a phase 1/2 trial (NCT04324996) investigating an IL-15 agonist combined with GM-CSF neutralizing single chain variable fragment (scFv), secreted from these chimeric antigen receptor NK cells expressing both NKG2D and ACE2, was conducted. Healthy NK cell infusions as above are under investigational development in another phase one study (NCT04900454). Some will hold promise for treatment of potential immune cell dysfunction beyond COVID-19 disease [326].

## 6. T Cells and the Adaptive Immune System in SARS-CoV-2 Research

### 6.1. Overview to T Cells and the Adaptive Immune System

T cells are thymus-derived lymphocytes that provide adaptive immune defense representing 56.6–84.6% of the B/T and NK cell populations [327,328]. The discovery of T cell CD4 (Leu-3 and T4) receptors occurred in the late 1970s, whereas a cluster of differentiation markers were clarified in 1984 at the first workshop on HLA molecules [329]. Further phenotypes thereafter included T_H_1, T_H_2, T_H_9, T_H_17, T_H_22, T_C_, and T_REGS_. T cell development occurs in bone marrow lacking characteristic CD4^+^ and CD8^+^ receptors (DN), undergoing SHM and selection processes that generate CD4^+^CD8^+^ double positive (DP) thymocytes or others that include CD4^+^ or CD8^+^ single positive (SP) thymocytes that emerge into the periphery as T cells. These generally exhibit a CD45RA^+^CCR7^+^ phenotype, although CCR7 is also expressed on DCs. The comparatively recent discovery of T_REG_ cells by Sakaguchi in 1995 defined with clarification that CD4^+^CD25^+^ cells express a nuclear transcription factor forkhead box P3 (FoxP3) that regulates development [322]. These also undergo clonal expansion where activation/differentiation occurs into effector T cells that mediate pathogen clearance, after which T cells undergo apoptosis, while memory T cells can persist with variable responses, with further data below to compare these with historical comparable standards of previous viral pandemics such as influenza, RSV, or smallpox. Cytotoxic CD8^+^ T cells are known to use a variety of proteolytic enzymes, including granzymes, while CD4^+^ T cells regulate maintenance through T_H_1 and T_H_2 lineages in concert with this CD8^+^ T cell response via regulation of exhaustion and antigen recognition receptors. T cells are further defined as naïve CD3^+^ CD4^+^ CD45RA^+^ CCR7^+^ (T_N_), which differentiate into central memory (T_CM_: CD45RA^−^CCR7^+^), effector-memory (T_EM_: CD45RA^−^CCR7^−^), stem-cell memory (T_SCM_: CD45RA^+^CCR7^+^CD95^+^CD122^+^), and peripheral resident memory (T_RM_ CD69, CD103, CD49) T cells [330]. T_RM_ cells produce key cytokines IFN-γ, IL-17, TNF-α, and IL-2, and these cells can express PD-1, LAG-3, and CTLA-4. In serum, CCR7 distinguishes T cell homing to LN when expressed, or effector memory (T_EM_) subsets migrating to tissues when absent. However, as we previously stated, DN B cells and DN T cells which do not express CD4 or CD8 are known to exist [331]. Below is illustrated all above current T cell classifications by cluster of differentiation marker (see Figure 11).

The TCR transcends T cell membranes, recognizing fragments of antigenic peptides bound to major histocompatibility complex (MHC class I/II) molecules in conjunction with other co-receptors. Similarly, TCRs undergo recombination during development to create a heterodimeric receptor. Most (95%) are composed of alpha and beta chains (α/β), but also gamma and delta chains (γδ) (5%), that transduce signals through receptor/peptide/MHC complexes located near the CD3 receptor which, again, has CDR regions. Therefore, TCRs bind with different affinities and utilize other T cell co-receptors (CD4/CD8) to affect cellular requirements via other cells affected by cytokines expressed with MHC class I or II antigenic peptide presentation to these T cells, and all are affected by cytokines [332].

### 6.2. Background to T Cells in Coronaviruses and Host SARS-CoV-2 Infection

Prior COVID-19 research on immunogens understandably focused on nAb responses, with less interest in overall cellular immunity. Interestingly, there is data accumulating which suggests that T cell responses are an important player in vaccine protection against chronic COVID-19 disease, even more so against recent variants of Omicron lineages (BA1, BA2, BA4/5, BQ1, and XBB) that display further epitope escape from recognition by nAbs. Epitopes represent the unique protein sequence that either viral antigens or vaccine immunogens stimulate recognition of, evoking protection against viral pathogens. These observations should have an impact for using current COVID-19 vaccines and for the development of next-generation vaccines against COVID-19 and other infectious diseases [333]. Early in the pandemic in 2020, case reports appeared across the globe (e.g., Italy) describing people with a rare disorder showing deficiency in antibody production or even no B-cells at all (e.g., agammaglobulinemia-XLA) [334]. Routine surveillance in this risk group identified two XLA patients who developed COVID-19 while receiving Ig infusions. These patients developed pneumonia which eventually resolved but never required ventilation or intensive care. This was the first time that B-cell response might be considered important, but it is not strictly required to overcome COVID-19 disease [335,336]. In addition, in 2020, groups in Germany and the USA independently discovered reactive T cells to the S glycoprotein during SARS-CoV-2 infection in around 30% of healthy donors when analyzing the T cell response of COVID-19 patients [337,338]. These T cells belonged to the CD4^+^ lineage and were primarily directed against C-terminal-epitopes of S protein, which has higher homology among S glycoproteins of hCoVs than the N-terminal region. Braun et al. further showed that these cross-reactive T cells were functional against the S protein C-terminal domain of the human endemic coronaviruses hCoV-229E and hCoV-OC43, as well as that of SARS-CoV-2 [338]. Daniela Weiskopf et al. mapped 142 T cell epitopes across the SARS-CoV-2 genome by using pre-pandemic blood samples to simplify the exact investigation of the SARS-CoV-2-specific CD4^+^ T cell repertoire. They demonstrated an array of pre-existing memory CD4^+^ T cells with cross-reactive capability with equal affinity to SARS-CoV-2 and the common cold coronaviruses hCoV-OC43, hCoV-229E, hCoV-NL63, and hCoV-HKU1 [337].

As research continued, all B and T cell markers CD3/CD4/CD8/CD19 were re-profiled as described above and below. Therefore, early indications of lymphocyte dysfunction (see Supplementary Data S5) elucidated this to show that T suppressor cell populations possessing the T cell activation marker CD28 required clarity. Furthermore, CD28 requires co-stimulatory molecules CD80 and CD86, of which CD80 is present on B cells, DCs, T cells, and Mϕ (requiring co-stimulatory molecules CD80/CD86). Hence, within chronic COVID-19 disease, and as there are changes within the T cell subsets, it is of note that, within T cell subsets, DN T cells exist that do not express classical CD4 or CD8 receptors. SARS-CoV-2 reactive T cells were found in peripheral blood and tonsil from donors unexposed to SARS-CoV-2, indicating that it is possible that antigens are present in other pathogens [339,340,341,342,343,344,345,346,347]. A number of these samples were analyzed by reports prior to the COVID-19 pandemic, eliminating the likelihood of exposure of the donors to SARS-CoV-2 infection [339,340,342,343,346,347]. An explanation to this observed phenomenon is that we are observing a recall response of SARS-CoV-2-cross-reactive memory T (T_MEM_) cells that were created upon encountering homologous proteins derived from other coronaviruses. Before SARS-CoV-2, six hCoVs were circulating with SARS-CoV, with MERS-CoV sharing the highest homology to SARS-CoV-2 in that it also caused respiratory syndromes [348]. Le Bert et al. even reported in blood samples from convalescent SARS-CoV-1 (>17 years ago) that T cells were detected which recognized SARS-CoV-2 [346]. Nevertheless, for SARS-CoV-1 and MERS-CoV, the dissemination was more restricted than for SARS-CoV-2, meaning that it is unlikely to find cross-reactivities over a wide range of the population. SARS-CoV-2 reactive T cells were found in peripheral blood and tonsils from donors unexposed to SARS-CoV-2 [339,340,341,342,343,344,345,346,347]. Though it is still heavily debated and unclear whether common cold hCoVs mediated this, cross-reactive T cells are of benefit in host defense against COVID-19 disease. Interestingly, in 20% of SARS-CoV-2-naïve donors and a higher percentage of infected or vaccinated donors, a report determined that a SARS-CoV-2 conserved peptide, S816-830, found in common cold hCoVs, activates CD4^+^ T cells, which led the authors to the conclusion that cross-reactive CD4^+^ T cell response might be protective against COVID-19 disease [349]. In contrast to these findings, a different group found the same epitope activated CD4^+^ T cells in more donors with breakthrough infections after vaccination compared to vaccinated donors with no breakthrough infection [350]. Moreover, it was reported by Bacher and colleagues that CD4^+^ T cells cross-reactive to SARS-CoV-2 in individuals not exposed to the virus displayed low avidity, which similarly correlates with individuals with severe COVID-19 disease [345]. These cross-reactive CD4^+^ T cells could be determined *in vitro* as responsive to SARS-CoV-2 proteins, leading to proliferation of the concomitant T cell clones stimulated by SARS-CoV-2. The relevance of these findings requires further investigation. On the other hand, four human coronaviruses (hCoV-OC43, hCoV-HKU1, hCoV-NL63, and hCoV-229E) circulate each year, causing the common cold. These four hCoVs share less homology with SARS-CoV-2. Indeed, it remains evident that cross-protective T cell epitopes seen in common cold hCoVs recognize SARS-CoV-2 [342,349,350,351]. Commensal bacteria as well as hCoVs can induce cross-reactive T cells to SARS-CoV-2 infection [352,353]. In COVID-19 patients, as well as in healthy controls, a public T_FH_ clonotype was detected that recognizes a SARS-CoV-2 epitope, S870-878 protein, and shares homology with a symbiotic bacterial antigen. Its abundance was found to be higher in patients with acute compared to chronic symptoms as compared to individuals with severe symptoms, leading to predictions that this clonotype has protective attributes [352]. Unexpectedly, the old vaccine strain BCG displayed homology with eight epitopes of NSP3 from SARS-CoV-2. In this case, Eggenhuizen et al. demonstrated that *in vitro* stimulation with SARS-CoV-2 proteins of BCG-primed CD4^+^ and CD8^+^ T cells led to enhanced cytokine production and proliferation in an HLA-dependent fashion. This mechanism might provide a partial explanation for the observation that BCG vaccination exerts some protection from COVID-19 [354]. Nonetheless, further studies to elucidate the exact features of cross-reactive T cells that could differentiate between SARS-CoV-2 infection and other pathologies will be required.

### 6.3. Helper T Cell Role during the Adaptive Immune Response to SARS-CoV-2

Historically, T cells were classified as T_H_1/T_H_2 and/or T_C_ types (CD4^+^ and CD8^+^), with the HIV discovery elucidating CD4^+^ receptor mediated entry some 30 years ago; however, between 2000 and 2014, further classification of chemokines as predominant homing ligands and receptors occurred, which was of key relevance to immune system regulation. T_H_1 cells secrete IFN-γ, IL-2, and TNF in comparison to T_H_2, where IL-4, IL-5, IL-6, and IL-10 are produced. CTLA4 (CD152) is preferentially expressed by T cells and regulates immune responses through interactions with B7-1 (CD80) and B7-2 (CD86) on antigen presenting cells (APC). A similar molecule, PD-1 (CD279), is also involved in immunoregulation through ligands PD-L1 and PD-L2. More recently, TIGIT has been found to regulate immune responses through interaction with CD112 and CD155 in a manner analogous to CTLA4. In contextual terms, the CD28 super-family of regulatory leukocyte receptors includes ICOS, CD28, CTLA4, PD-1, and BTLA, of which CD28 is a co-stimulatory T cell activation molecule for B cell ligands CD80 and CD86, compared to PD-1 as an inhibitory receptor for PD-L1/PD-L2. Recently, it has been observed that immunocompromised cohorts with immune-related pneumonitis possess more CD4 T_H_2 cells expressing CCR7, but also that CXCR3^+^GATA3 is preferentially expressed [355]. Initially, it was proposed that immunity could be divided into three types, in short, initially, IFN-γ producing cells (NK cells, T_C_ and T_H_1) regulating activation of monocytes; secondly, T_C_ and T_H_2 regulation of mast cells, basophils, and eosinophils through IgE; and a third type mediated by RORγt^+^ T cells and T_H_17 regulation of monocytes and neutrophils [70,356]. Early in 2020, a noted marked reduction in T cells was seen in laboratory studies (n = 552) in SARS-CoV-2 infection with CD4^+^CD8^+^ cell counts (75.95%:71.54% respectively) during COVID-19 disease, accompanied by upregulation of PD-1 with suggestions that IL-6, IL-10, and TNF-α were not produced by T cells [357]. This was a key finding, suggesting that cell dysfunction, as PD-1 (CD239), is an inhibitory receptor that regulates both chronic viral infection and cancer through affecting T cell apoptosis pathways. This dysfunction occurs via binding of PD-1 to PD-L1 and PD-L2 on APCs and effectively controlling IFN-γ cellular release. In addition, lymphocyte activation gene 3 (LAG-3), together with T cell Ig and mucin domain 3 (TIM-3) pathways, were upregulated with concurrent sCD28, sCTLA-4, sBTLA, CD270, and sCD80 increases [358,359]. Comparatively less is known about LAG-3, although it is not present on naïve T cells and is constitutively expressed on T_REGS_ implicated with T cell exhaustion. LAG-3 presents on DN T cells and intraepithelial lymphocytes, γδ T cells, and NK cells, and therefore remains a focus of cancer research as a checkpoint protein [360,361]. CD28 concurrent reduction and shedding could have significant effects on B lymphocyte signaling through respective ICOS ligands, and all of these would usually be expressed within the GC of primary lymphoid organs during follicular development. As discussed above, DC subtypes form the core of immune response cells through expression of MHC class II and changes to subtypes within SARS-CoV-2 infection, suggesting that systemic cDC2 cells undergo changes that could affect the maturation and priming of T_FH_ antibody responses [362]. Furthermore, during infection, individuals with COVID-19 disease displayed a higher expression of T cell Fas (CD95) and sCD95 in CD4^+^ T cells, but also PS correlated with T cell counts indicating an overall increase in T cell apoptosis and increases in caspase 1/3, and this intracellular activation within both CD4/CD8 T cells was confirmed [363]. Notably, other studies indicate that PS receptors can be associated with increases in infection rates, with increased CD95 implicated in the regulation of auto-reactive T cells [364].

The Gil-Mansos group recently conducted a comprehensive FACS analysis comparing a control vs COVID-19 disease group (n = 51) within recovered COVID-19 individuals to demonstrate a significant cell cluster at 10 months after recovery of CD4^+^ CD45RA^−^ CCR4^−^ CCR10^−^ CD27^+^ CCR6^−^CXCR3^+^ CD127^+^ cells. Their analysis inferred a response within circulating T_FH_1, PBs, and the follicular-dendritic-cells (foDC) axis [17]. In this report, it was found that T_FH_ cells within the SARS-CoV-2 infected group expressed both ICOS and PD-1, which is relevant to normal responses occurring outside the GC that could affect T_FH_/B cell lymphocyte development [10]. Therefore, a potential imbalance within T_CM_ cells during acute COVID-19 was seen to occur, which polarizes T_FH_ lineages leading to potential dysregulation of the B cell response outside GC. This was quantified to find the CD4^+^ T cell’s tendency to T_H_2-like phenotypes in chronic SARS-CoV-2 infection that increases in CD45RA^+^CD62L^−^ (T_CM_) and CD45RA^−^CD62L^−^ (T_EM_), compared to the decreases in T_CM_ CCR6^+^ T_H_17-like cells [365]. Through stimulation of these T_FH_ cells by agonism of the ICOS receptor that would produce normal synthesis of IFN-γ/IL-21, it was observed in samples from hospitalized COVID-19 subjects that T_FH_ cells (CD3^+^ CD4^+^ CXCR5^+^ ICOS^+^) within ambulatory subjects encompassed, on average, 15% T_FH_ of the peripheral blood CD4^+^ compartment relative to the normal 8% T_FH_ population [366]. Vanderbilt University’s recent single cell sequencing study expanded on the importance of this cell subset in a healthy cohort (n = 10) where analyses further showed functional T_H_ cells as CD4^+^CD38^++^ICOS^++^Ki67^+^ (but also expressing CTLA4, CD44, CD45RO, CD127, and others) alongside key chemokine homing receptors CXCR3, CXCR5, and HLA-DR, confirming that cell signaling receptors were expressed and that the T cell proliferation marker Ki67 was working normally in T_N_ subsets [367]. Epitope determinants of SARS-CoV-2-specific CD4^+^ T cell lines reveal SARS-CoV-2 M protein-driven dysregulation of ISG in chronic COVID disease (ISG15, IFITM1, IFI16, MX1, STAT1, OAS1, IFI35, IFIT3 and IRF7) [368]. The importance of a T_H_1 response cannot be underestimated in immunogen development and is historically thought to be the identifying factor producing IFN-γ, IL-2, and TNF to evoke cell-mediated immunity and phagocyte-dependent inflammation. Current *in vitro* and in clinical settings research is indicative that, within the CD4^+^ T_FH_ compartment, CXCR3^+^ expression is upregulated in acute cases and then downregulated with severity in COVID-19 disease in PB cells, and also that CD4^+^ T cells expressing PD-1^hi^ do not express CXCR5 [162,369,370,371,372]. As we discussed, the dynamics of each cell type can only be identified by individual markers. Therefore, given the above, it is apparent that, in case control studies (n = 27) in other inflammatory conditions, such as RA, where autoantibodies affect homeostasis and TNF-α, production seems to localize with specific particular epitopes on T cells near the citrullinated vimentin co-receptor that also potentially SARS-CoV-2 utilizes [12,285,374]. This has been observed to be blocked by utilizing anti-TNF therapeutics under research investigation. Interesting, this study measured migratory T_H_ CD4^+^CD45RA^+^ cells expressing CD62L^+^CD95^+^ that can be downregulated or shed, respectively, in dysregulated similar pathologies [285,371,372].

### 6.4. Cytotoxic T Cell Role during the Adaptive Immune Response to SARS-CoV-2

Cytotoxic CD8^+^ T cells (T_C_) are major killers of pathogens and neoplastic cells, with CD4^+^ T cells playing important roles in the maintenance of the CD8^+^ response and the prevention of exhaustion that utilizes a variety of mechanisms. Like above, CD8^+^ T cells differentiate into effector cells and produce IFN type I with Mϕ and DCs. Other cytokines, such as TNF-α, IL-15, and IL-18, facilitate CD8^+^ T cell response. T_C_ cells, for example, are generated in the presence of IL-2 and IL-12 and acquire the ability to secrete cytokines such as IFN-γ. Cytotoxicity of T_C_ cells is determined by granular enzyme release, such as granzyme B and perforin, with virulence toxicity varying between cytotoxic T cells depending on cytokine stimulation, but also transcription factors implicated in T_C_ generation, including T-bet, blimp-1, IRF-4, and GATA-3. Illustrated below (see Figure 12) are interactions of T_C_ cells and relevant cytokines. As we discussed, it can now be seen that further information is available on these cells, which are decreased in immunocompromised cohorts with immune-related arthritis that have lower levels of CD8 T_CM_ cells [355].

In SARS-CoV-2 infected patients, it was seen that T cells expressing CD28 could be downregulated, but CD8^+^ T cells also express PD-L1 and CXCR3^+^, potentially also significantly associated with survival in SARS-CoV-2 infection alongside N protein specific CD8^+^ cells [373]. As before, groups in Holland saw enrichment of CCR6^+^ CCL20^+^ within CD8 T cells during COVID disease (n = 22) in BALF samples compared to serum, with fewer T_H_1 cells indicating preferential differentiation and migration of this subset in severity into the lung, but also an impaired T_H_ cell response [374]. During SARS-CoV-2 infection, there was also a significant increase in terminally differentiated T_EM_ cells (T_EMRA_) and T_EM_ cells localizing with T_CM_ (CCR7^+^ CD45RA^−^) differentiating into T_H_2-like secreting IL-5, with T_CM_ cells expressing chemokines (CX3CR1, CCR6, CXCR6, and CXCR3 that represent lung-homing receptors). T_CM_ cells could secrete TNF-α, but also SARS-CoV-2 CD4 S-protein-specific T cells displaying a T_CM_ phenotype with specific CD8^+^ cells heterogenous towards T_EM,_ T_EMRA,_ and T_SCM_ [155,370,373]. Therefore, as lymphopenia can occur and cause potential insufficient antibody responses in SARS-CoV-2 infection, it is necessary to look at dysfunction inside and outside GCs in COVID-19 disease [155,370,375]. Reports are conflicting around CD3^+^CD8^+^ cells expressing CD38 that downregulate MHC class II (HLA-DR) during SARS-CoV-2 infection, although others indicate that this correlates with disease severity [376,377]. In some cohort studies within immunocompromised patients, it does, however, appear that different immunogens evoke individually different responses that could vary by underlying comorbidity. For example, healthy controls would express B cells (CD38^+^CD19^+^) and T cells (CD8^+^ HLA-DR^+^) three weeks after two doses of vaccine (n = 42); in comparison to transplant patients, little change was seen in cells expressing HLA-DR^+^, CD38^+^, and PD1^+^, but in immunocompromised cohorts, 13.3% produced an IgG S-protein-specific response [378]. This single cell analysis showed changes, or rather increases, in CD8^+^ T_EM_/T_EMRA_ cells [299]. More recently, in a yet to be reviewed pre-print, elevated or prolonged CD8^+^ activation was seen to be accompanied by elevations in IL-4, IL-7, IL-17, and TNF-α [379]. Furthermore, this was accompanied by CD8^+^PD-1^+^CXCR3^lo^CCR6^+^ T cells in severity in the lung, which suggested a reduced cytotoxic antiviral function of cytotoxic T cells [374]. Recent, yet to be peer reviewed, suggestions have been made that, in severity compared to moderate illness, the CD4 CD8 dysregulation at 3 months is not related to the cytokines produced that are predominant but that affect each of the T cell compartments through IL-4, IL-7, IL-17, and TNF-α [379]. Therefore, as immunogenic proteins are seen to affect the adaptive immune response, it was also quantified that although 86% of individuals possessed antibodies against S2 protein, more were produced against RBD, and that T_C_ epitopes at specific amino acid residues (884–891 and 1116–1123) were identified that could be responsible for cross-reactivity, with some indications that this was with another hCoV (OC-43) [130].

### 6.5. Regulatory T Cell Overview during the Adaptive Immune Response

CD4^+^ Foxp3^+^ regulatory T cells (T_REGS_) are a central component of immune regulation, as humans are reliant on T_REGS_ expressing Foxp3 gene, which has been extensively investigated in the context of cancer regulation and COVID-19. Follicular T_REGS_ (T_FR_ cells) can inhibit antibody production, whereas follicular T_H_ cells (T_FH_ cells) stimulate it. T_FR_ cells are found in blood; however, comparatively less is known about the developmental signals. T_REGS_ represent a fraction of total CD4^+^ T cells in sera and tissue, which constitutively express CD25 that can be shed and is part of the IL-2 receptor. Nearly all laboratory case studies indicate that current therapeutic COVID-19 remedies do in fact act to increase this subset, with several molecular mechanisms proposed. T_REGS_ constitutively expresses high affinity IL-2 receptors and scavenges the T cell growth factor IL-2, thus preventing suppressor actions on proinflammatory T cells. T_REGS_ also express a surface receptor molecule CD39 (ENTPD1ATP), which hydrolyses ATP within and outside cells. In notable disorders such as autoimmune thyroid conditions (e.g., GD), where prevalence estimates are 2–5% of the population, the role of T_REGS_ is likely to be clearer, as it was seen that in untreated autoimmune thyroid diseases (AITD) there was a clear reduction in T_REGS_ (RORγt/Foxp3) [380]. Autoimmune thyroid diseases (AITDs) also involve immunologic dysregulation, with the presence of cell and humoral immune responses against thyroid gland antigens, T and B cell infiltration, and autoantibody generation; they are among the most common autoimmune disorders. AITDs include Hashimoto’s or chronic lymphocytic thyroiditis, which is a hypothyroid condition, and GD, a hyperthyroid condition. AITDs emerge via epigenetic and gene-environment interactions, and affected individuals are at risk for additional autoimmune diseases through associated genes, including immunoregulators (e.g., HLA, CTLA-4) and thyroid specific components (e.g., TSH receptors, thyroglobulin, and others). Individuals with immune-related thyroiditis have more CD4 T_H_17 cells at baseline [355]. T_H_1, T_H_2, T_H_17, T_H_22, and T_H_9 lymphocytes are involved in AITD development, as well as T_REGS_ cells. Large scale, real-world studies are beginning to establish links between other disorders such as asthma and the risk of AITD. Such studies should provide information to optimize clinical guidance regarding how to diagnose and treat people with autoimmune disorders as we further unravel the complex endocrinological and inflammatory interactions and mechanisms that are involved [381,382]. T_REGS_ function, as above, can additionally be seen in DM1 patients where impairment of selected chemokines CCL3 and CCL4 has been suggested to impair adaptive T cell suppressive responses [383]. Case studies are contrasting, with respect to T_REGS_ cells to date, with some indicating that T_REGS_ increase with COVID-19 severity and others indicate a decrease in quantity [384]. A notable recent study (n = 19) examined these CD3^+^CD4^+^CD25^+^CD127^low^ T cells up to 3 months after SARS-CoV-2 infection to find that IL-10 and TGF-β were produced upon stimulation with either S1 or N protein, and, importantly, that CD8^+^ cells were producing IFN-γ, as all 3 cytokines are required for suppression, proliferation, and anti-viral activity [385]. However, recently, studies on a novel cell type MDSCs, although not conclusive, are emerging to elucidate that, far from being clear, implicate TGF-β in COVID-19 MDSCs as suppressing CD4^+^ CD25^+^ Foxp3^+^ cells and evoking CD4^+^ CD25^−^Foxp3^+^ expansion, affecting T_REGS_, the autoreactivity of T cells, and suppressing IFN-γ [187,386]. The differences between T_FR_ and T_REGS_ have been examined prior, in order to observe whether CD25 signaling is independent or dependent on IL-2. Clarification of blimp-1 and BCL6 elucidated that CD25^−^ T_FR_ cells lose part of their IL-2-dependency in T_REGS_ in exchange for enhanced expression of BCL6 and other T_FH_-related genes with CXCR5 expression [387]. T_REGS_ have also, interestingly, played a role through chemokine research developments in preventing bowel inflammation in colitis patients by suppressing cytokines related to T_H_17 [388,389,390,391,392].

### 6.6. T_H_17 Cell Overview during the Adaptive Immune Response

The complete cellular relationship between T_H_17 and T_REGS_ cells is comparatively clearer, as both appear to have a symbiotic relationship in maintaining regulation [388]. It is believed that this occurs through T_H_17 transcription factors STAT3 and RORγt, which regulate host cell proliferation or suppression. T_H_17 cells are characterized by cytokine secretion (IL17A, IL17F, IL21, and IL22), together with receptors (CD161, CCR4, and CCR6) [393]. They are also considered to be regulated by DCs through IL-6, IL-1β, TGF-β and IL-23, which, in turn, polarize towards T_H_17 cells that differentiate into one of four or more phenotypes, T_H_1, T_H_2, T_FH,_ or T_REGS_ cells expressing the IL-23 receptor, thereby being responsive to DC IL-23 secretion [394]. For example, in IBD, excessive IL-17 secretion may cause excessive cell proliferation and differentiation, and similarities with IL-1 expression in mucosa between individuals affected by either Crohns or UC, with expression of CCL3 CCL4, CCL5, CCL7, CXCL5 CXCL8, CXCL10, CCL20, CXCL5, CXCL8, and CXCL10 in epithelial compartments with changes in other chemokines that bear similarities to post-acute sequelae of COVID-19 (PASC) [395,396,397,398,399,400]. Due to the dysregulation, or rather, upregulation, of T_H_17 and T_H_1, with a combination of reduction in the number of T_H_2 and T_REGS_ and imbalances in the immune system in SARS-CoV-2 induced COVID-19 disease, the levels of inflammatory cytokines and chemokines changes occurring, such as CCL3, GM-CSF, TNF-α, IFN-γ, G-CSF, IL-2, IL-8, and IL-1β, are increased in localized tissues, leading to cytokine release syndrome (CRS), inducing further damage to the respiratory system with concurrent changes to vascular permeability [389]. Early in the COVID-19 pandemic, researchers observed a skewed and increased ratio of T_H_17 cells in individuals, in severity (n = 80) of this order: T_H_17/T_REGS_, RORγt/Foxp3, and IL-17/IL-10. This illustrates not only increased T_H_17 cells, but that suppression occurred of T_REGS_ and IL-10 with less Foxp3 expressing T_REG_ cells [401]. More recently, it has been confirmed, in Egypt, in a pediatric cohort of mixed COVID-19/MIS-C individuals, (n = 31) that IL-17A was upregulated [402]. Therefore, given that this is consistent, it appears as though SARS-CoV-2-T_REGS_-induced COVID-19 disease can be associated with a decrease in T_REG_ cells and an increase in T_H_17 cells. However, different T cell populations, as above, are present in deceased COVID-19 patients and in improved patients. Disease progression in severity seems to reflect this (n = 7) compared with improved patients (n = 23) that had higher T_H_1, lower T_H_2, higher T_H_1/T_H_2 ratio, lower IL-4, higher IFN-γ, higher IFN-γ/IL-4 ratio (d), lower T_REGS_, higher T_H_17, higher T_H_17/T_REGS_ ratio, lower IL-10, higher IL-17, higher IL-17/IL-10 ratio, and higher depleted T cells. It has been suggested that the T_H_17-IL-17A axis may be a plausible therapeutic target for COVID-19 that could be researched to investigate in clinical trials. In PBMCs from a COVID-19 individual, T_H_17 cells were significantly increased, leading to a cytokine storm, which, in turn, accelerated disease progression. Their results imply that overactivation of T_H_17 cells and elevated CD8^+^ T cell cytotoxicity may partly explain the severe immune damage in these patients alongside immune complex formation [403]. Newer clinical trials are now starting to trial allogeneic CD45RA^−^ T_MEM_ therapy in adults affected by COVID-19 pneumonia in phase 2 trials with SARS-CoV-2 specific T cells, with early promise indicating a concurrent increase in NK cell activity (NCT04578210) [404].

### 6.7. γδ T Cell Overview during the Adaptive Immune Response

Gamma-delta (γδ) T cells are “unconventional” T lymphocytes that do not require antigen major histocompatibility complex (MHC) presentation. There are two main subsets of human γδ T cells that are classified according to the expression of the δ chain of the T cell receptor (TCR) [405]. Vδ1 T cells are more common in mucosal tissues and are involved in the first line of immune defense against solid tumors and infections. Vδ2 T cells, a subset uniquely associated with the Vγ9 chain (termed Vγ9Vδ2), are abundant in the peripheral blood and play an immune effector role in tumor surveillance as well as in antimicrobial defense [406]. Vγ9Vδ2 T cells have several initial roles, including directly killing infected cells by different mechanisms, and to interact, prime, and modulate the functions of other innate and adaptive immune cells via cytokine secretion, antigen presentation, and cell contact to develop antimicrobial immunity [407]. The mechanisms of phosphoantigens (pAg) recognition by Vγ9Vδ2 T cells involve the butyrophilin (BTN) protein family. Butyrophilin 3A1 (BTN3A1, CD277), expressed by both immune and somatic cells, that directly binds to pAg intracellularly via its cytoplasmic domain B30.2, results in a conformational change in its ectodomain that is detected by Vγ9Vδ2 T cells [408,409]. BTN3A1 interacts at the plasma membrane with another BTN family member, BTN2A1, which is a direct ligand for the Vγ9 TCR chain, thus providing synapsis between Vγ9Vδ2 T cells and target cells [410]. Several studies have confirmed that Vγ9Vδ2 T cell activation is dependent on BTN3A during infections [408]. Recently, it has been shown that the γδ T cell response to influenza changes throughout the human lifespan [411]. Whereas the primary response was by IFN-γ-producing Vγ9Vδ2 T cells, the γδ T cell repertoire was markedly different in neonates and adults. The neonatal γδ T cell repertoire showed much higher diversity, whereas the adult repertoire was strongly Vγ9Vδ2 dominant. Sant et al. further identified vulnerability to influenza viruses in newborn and elderly donors lacking Vγ9Vδ2 TCR. This shows that γδ T cells appear to be strongly involved in respiratory viral infections such as influenza viruses. The importance of IFN-γ producing Vγ9Vδ2 T cells, commonly found in adult peripheral blood, is particularly striking. Their importance in these different viral infections indicates a strong importance of this subtype in anti-viral immunity. There appears to be a direct link between the anti-viral capabilities of γδ T cells against SARS-CoV-2 infection. After the SARS-CoV-1 outbreak in 2003, Poccia et al. found an increase in γδ T cells in survivors 3 months after infection [412]. Specifically, this increase was observed only in the Vδ2 subtype that is commonly most found in sera. It was also shown that Vγ9Vδ2 T cells isolated from blood sera were able to significantly reduce viral load in *in vitro* studies. It was demonstrated that Mϕ infected with the variants showed a different effect on γδ2 T cells whose antiviral properties are promoted by Mϕ. In a recent, not yet peer reviewed, report, it was indicated that activated γδ2 T cells elicit strong cytotoxic and non-cytolytic anti-SARS-CoV-2 activities *in vitro* in response to the original SARS-CoV-2 characterized [413]. Using an *in vitro* co-culture model, the impact of Mϕ infected with SARS-CoV-2 variants on γδ2 T cell activation in response to each variant of concern was highlighted, and infection with the γP.1 (Brazil) and β-B.1.351 (South Africa) variants resulted in the highest secretion of TNF-α by γδ2 T cells [414].

## 7. Autoantibodies in SARS-CoV-2 Research

Autoantibodies in SARS-CoV-2 infection remain under investigation. Recent reports (n = 987) from Bastard et al. showed that 10.2% of those who had chronic COVID-19 disease did indeed possess autoantibodies against type I interferons (mostly interferon IFN-α2 or other type I IFN) compared to the control group, which concurs with other studies [415]. However, in epidemiological studies prior to 2020, larger cohort studies (n = 8972) indicated that a high rate of base-line incidence prevails regardless of autoantibody occurrence [416,417]. In addition, autoantibodies to IL-1RA were also present in 62% (13/21) of samples of multisystem inflammatory syndrome in children (MIS-C), and further research is required to see if these have pathological effects [418]. Other studies awaiting peer-review notably observed significant increases in autoantibodies (n = 30) in severity to desmoglein 2 (DSG2), that can be present in heart, skin, or skeletal muscle [419]. The significance of autoantibody changes, presently, is largely unclear in research, and other authors concur that this could be a transient change but would need further research [420].

## 8. Limitations

There is a requirement to acquire more data to sharpen the clarity of understanding of cellular and humoral immunity on a global scale, particularly where SARS-CoV-2 infection has highlighted areas of knowledge gaps in research and clinical settings. As the 2019 coronavirus is a newer pathogen, extensive research studies and clinical trials have occurred with diagnostics also evolving. As one measure, debate is ongoing concerning whether swabbing both the nose and throat increases PCR test sensitivity to any pathogen; most rapid antigen test manufacturers recommend nasal swabs via lateral flow devices, while global public health authorities in the past suggested PCR nose and throat, as applicable to other infections. Additional studies concerning viral antigen expression within a host, shedding, and longevity in concert with immune responses require both single cell analyses or immunohistochemistry amongst other laboratory techniques to optimize and validate diagnostics and therapeutics for any pathology, to ascertain accuracy of swabbing of nose or throat (see Supplementary Materials). Variabilities include both PCR cycle threshold and tissue sample. Mast cells, basophils, and eosinophils have not been specifically covered in this review. In the transcriptome studies, limitations are recognized within UMAP as skewing variable gene and Ig constant regions. Co-existing medical conditions or other viral or microbial cellular environments also vary within hosts due to an array of factors including gender, age, geographical location, genetics, and health status prior to, during, and post SARS-CoV-2 infection. Thus, revisiting and expanding on this review in future times would hold value.

## 9. Discussion

SARS-CoV-2 and other viruses evolved as zoonotic infections that can cross animal barriers. It is necessary to consider that, regardless of the origins of SARS-CoV-2, there was genetic homology of 96.5% with *Homophilus affinis*, and cellular recombination events are necessary for both an immune response and continued viral propagation within animal hosts. Currently, variant surveillance data on SARS-CoV-2 variants in other animals includes monitoring in different animal populations such as mink (1320) and bats (8), with comparatively less monitoring in pet and zoo animals (see Supplementary Data). Records suggest that only two pathogens have largely been eradicated in humans and animals, which are smallpox (an *Orthopoxviridae variola*) and rinderpest (a *Paramyxoviridae morbillivirus*), respectively [421]. Therefore, further consideration of current surveillance within and across animal populations occurs in order to monitor other potential pathogenic coronaviruses (e.g., infectious bronchitis in birds, porcine delta coronavirus, and feline coronavirus) to prevent such zoonotic infections recurring in the future. Experimental research between the 16th and 18th century throughout the world, begun by Edward Jenner in the late 1700s on cowpox, eventually led to modern day interpretation and use of the word “vaccine,” referring to the substance being derived from vaccinia (cowpox). The smallpox pandemic caused by variola virus was eventually declared eradicated in 1980 by the World Health Organization, through research development utilizing what some would consider the pioneering vaccine immunogens by which standards are measured. Many other vaccine immunogens now exist, with research development improvements summarized here that illustrate the goals and research of thousands of people across the world. Estimates of overall smallpox immunity are around 50 years, largely due to pioneers in this field that also include Ehrlich, Medawar, and Edelman, and many others whose research into syphilis, actively acquired tolerance, and the structure of antibody molecules laid the foundations for Kohler and Milstein’s discovery of how to produce monoclonal antibodies.

A crucial and partially resolved question involves whether SARS-CoV-2 (COVID-19) disease results in long-term protection in humans. Analyses of B cell responses up to one year after infection show that there is a quantifiable neutralizing antibody response with memory B cells against both N and S protein in most recovered COVID-19 patients, which are stable or degrade slowly. Throughout the above case studies, virus evolution and future vaccine immunogen development can affect physiological cellular immune responses, and our knowledge is only now becoming clearer. Interestingly, one study examined interaction and correlation of gene expression within immune cells in a small cohort. Genetic susceptibility factors within the immune response show initial correlations, as we discussed, within B cell Ig chains (IGLC3, IGHG3, JCHAIN, and CD27), CD8^+^ T cells (GZMA and CCL5), and cDC2 elongation factors (EEF1A1, EEF1B2, EEF1G, EIF3L), with monocytes/Mϕ and cDC2 gene protein changes (IFI27, IFITM1, IFITM3, IFITM2, IFI30, and OAS1) [422]. Current SARS-CoV-2 evolution through S1 protein mutations appear to enhance re-infection from initial Omicron variants (BA1, BA2, BA4/5, BQ1.1, and XBB derivatives) through retaining known B cell escape epitopes that include L452R, F486V, and R493Q, and through acquiring other similar mutations (R346, N460, K444, and N460) in derivative lineages, which are known to cause issues with current specific monoclonal antibody therapeutics [423,424]. Other researchers have clarified details on the pipeline of current monoclonal therapeutics [425,426]. With regard to T cell responses, epitope analyses and characterizations have largely occurred, and can be seen below on either database (Supplementary Materials).

## 10. Conclusions

After extensive research, we have quantified and outlined the nature of current specific receptors and proteins relevant to clinical laboratories and medical research by documenting both innate and adaptive immune system cells within current coronavirus immunology data and other pathologies to date. B cell generation of antibodies, followed by neutrophils in pathophysiology, releases initial cytokines and correlates that are dependent on antigen-presenting cells of monocyte, macrophage, and dendritic cell lineages. However, each of these relates to defined T cell lineages. There are evidential increases in not only S protein immunogen responses, but also to N and M proteins. In this article, we considered cellular markers according to current immunological literature and dictations from experts in the field. Many senior scientists between April and September 2020 wrote letters in this regard (see Supplementary Materials) that demonstrated overall infection antibody positivity of 23% (NY), 18% (London), and 11% (Madrid) are dependent on the other 10 or more T cell subtypes performing all the regulatory functions of the immune system, which are arguably more important in all pathologies [427,428,429,430,431,432,433,434,435,436,437,438,439,440,441,442,443,444,445]. To conclude this review, we hope that the biomolecular information provided is prioritized in clinical and therapeutic trials, and expanded upon to better ensure patient care, therapeutic development, transmission, and mortality rates are improved world-wide, to more accurately target COVID-19 and similar pathologies that also host dystopian properties, and the details within will provide further information for all future researchers.

## Figures and Tables

**Table 1:** Antibody isotypes concentrations in sera and complement activation ability [83]. Designed and readapted with legal permission site accessed on 12 January 2023 from source (https://www.thermofisher.com/rs/en/home/life-science/antibodies/antibodies-learning-center/antibodies-resource-library/antibody-methods/introduction-immunoglobulins.html accessed online: 3 February 2023). (2022, Thermofisher). For further details see Thermo Fisher Scientific.

Isotype	Serum Level (mg/mL)	Molecular Weight (Monomer)	Complement Activation	Half Life
IgA1	0.6–3	160 (monomer)	–	5.5
IgA2	0.06–0.6	160	–	5.5
IgM	1.5	970	+++	5–10
IgE	5 × 10^−5^	188	–	2
IgG1	3.8–11.4	146	++	23
IgG2	1.5–6.9	146	+	23
IgG3	0.2–1.7	165	+++	7
IgG4	0.08–1.4	146	–	23

Note: Estimated Serum Level Concentrations, Molecular Weight (kDa), Complement Activation: – no, + yes, ++ strong, +++ very strong, Half Life (Days), (see Supplementary Materials).

**Table 2:** Frequency of Individual Serological Response During SARS-CoV-2 Infection (%) [127]. Copyright permission refer to citations and/or Supplementary Data.

Antigen	Mild			Moderate			Severe			Total		
	IgG	IgA	IgE	IgG	IgA	IgE	IgG	IgA	IgE	IgG	IgA	IgE
Spike (FP)	94.7	16.7	66.7	100	38.5	92.3	100	54.5	90	97.3	30.1	79.7
Stable Spike Trimer	97.4	25	12.1	100	42.3	50	100	54.5	60	98.7	35.6	33.3
RBD	92.1	22.2	0	100	42.3	3.85	100	63.6	10	96	35.6	2.9
S1	89.5	22.2	0	100	42.3	34.6	100	63.6	20	94.7	35.6	15.9

**Table 3:** B cell phenotypes (adapted from Li et al.) [107]. Copyright permission refer to citations and/or Supplementary Data S2, also see Figure 3.

B Cells	Markers			Chemokines	Ig	Amount	
Naïve B Cells	CD27^+^	ND	ND	ND	IgD^+^	29–69	[106,113,114,115,116,117,118]
Unswitched B Cells	CD27^+^	ND	ND	ND	IgD^+^	5–22	[114]
Switched B Cells	CD27^+^	ND	ND	ND	IgD^−^	8–43	[114]
B Cell DN1	CD27^−^	CD21^+^	CD11c^−^	CXCR5^+^	IgD^−^	4–6	[107,115]
B Cell DN2	CD27^+^	CD21^−^	CD11c^+^	CXCR5^−^	IgD^−^	ND	[116,117]
B Cell DN3	CD27^−^	CD21^−^	CD11c^−^	ND	IgD^−^	ND	[118]
B Cell DN4	CD27^−^	ND	ND	ND	IgD^−^	ND	[118]

Note: ND= no data, Amount (Estimated total B cell %), also see Figure 3 or Supplementary Data S2.

**Table 4:** Functional diversity of dendritic cells.

	pDC	cDC1	cDC2	moDC	Tissue DC
**Cellular** **Differentiation Marker**	CD11c^−^	CD11c^++^	CD11c^++^	CD11b/c^+^	CD11c^lo/hi^
	CD303	CD141^+^	CD1c^+^	CD1c^+^	CD1c^+^
	CD123	CD370 (CLEC9)	CD172a^+^ (SIRPα)	CD1a^+^	CD1a^+/−^
	CD45RA	CD14	CD14	CD206/CD209	CD14^+/−^
		CD45^+^ CD103^+^	CD5^hi/lo^ CD103^+^ CD123^−^	CD172a^+^ (SIRPα)	
**Cytokine/** **Chemokine**	IFN–α, CCR7	XCR1			

## Data Availability

All related data is presented in this paper. Additional inquiries should be addressed to the above or corresponding author(s) attached or via enquiry at www.biochem123.org or info@biochem123.org (accessed on 23 January 2023).

## Supplementary Materials

The following supporting information can be downloaded at: https://www.mdpi.com/article/10.3390/vaccines11020408/s1. **Additional Data S1**–**S5**, **S1**—Adhesion Molecules changes during SARS-CoV-2 infection, **S2**—B cells Classification; **S3**—Overall Frequency of Individual Antibody Responders against SARS-CoV-2 antigens; **S4**—Chemokines in Immune Cell Migration, **S5**—T Cell Markers. The following supporting information can be viewed or downloaded at: T cells—https://www.biospace.com/article/60-scientists-sign-letter-petitioning-fda-for-t-cell-recognition/, accessed on 23 January 2023, Office of National Statistics COVID-19 Infection Surveys Coronavirus (COVID-19) Infection Survey, UK, Office for National Statistics, Nuffield Department of Medicine: Results-antibody positivity updates from ONS—Nuffield Department of Medicine (ox.ac.uk, accessed on 18 January 2023): Swab the Throat as Well as the Nose? The Debate Over the Best Way to Test for SARS-CoV-2|Infectious Diseases|JAMA|JAMA Network and Understanding At-Home OTC COVID-19 Antigen Diagnostic Test Results | FDA; COVID-19 variant surveillance—covSPECTRUM (cov-spectrum.org, accessed on 18 January 2023); Animal SARS-CoV-2 surveillance COVID19 DASHBOARD ANIMALS GLOBAL and Tableau Public SARS-ANI VIS (csh.ac.at, accessed on 18 January 2023), Clinical Trials—Home-ClinicalTrials.gov, Human Leukocyte Antigen Information—COVIEdb—A database for potential immune epitopes of coronaviruses (zju.edu.cn, accessed on 18 January 2023), IEDB.org (accessed on 18 January 2023): Free epitope database and prediction resource, COG-UK/Mutation Explorer (gla.ac.uk, accessed on 18 January 2023) Flow cytometry (FACS) staining protocol (Cell surface staining) Yale Flow Cytometry Bayesian approach to single-cell differential expression analysis|Nature Methods Enzyme-Linked Immunosorbent Assay (ELISA)|SpringerLink. For further details see U.S. Food and Drug Administration (fda.gov, accessed on 18 January 2023), European Medicines Agency|(europa.eu, accessed on 18 January 2023) or Medicines and Healthcare products Regulatory Agency—GOV.UK (www.gov.uk, accessed on 18 January 2023).

## Abbreviations

IRF4/7 Interferon regulatory factor(s); IFI44L Interferon induced proteins; IFIT1 Interferon-induced protein with tetratricopeptide repeats 1; IFIT1 Interferon-induced protein with tetratricopeptide repeats (IFIT3); STAT1/6 Signal transducer and activator of transcription 1/6; CASP3/8/10 caspase 3/8/10; ISG15 Interferon Inflammatory signature Gene 15; Interferon-inducible transmembrane IFITM; interferon-induced murine MX; 2’-5’-oligoadenylate synthetase OAS1; Ig Light and Heavy Chains or Joining IGLC3, IGHG3, JCHAIN; Granzyme A GZMA; Elongation Factors EEF1A1 (also EEF1B2, EEF1G, EIF3L); THBS1, THBD Thrombospondin; ALOX5AP Arachidonate 5-lipoxygenase activating protein, Receptor tyrosine kinase AXL and CLU gene (clusterin). PU-1 Transcription factor; ZEB2 Zinc Finger E-Box Binding Homeobox 2; MTG16 myeloid translocation gene; TCF4 Transcription Factor 4; BCL11A, RAGE receptor for advanced glycosylation end products. IRF Interferon regulatory factor. GZMA Granzyme A, Ras homolog gene family, member B (RHOB). All other abbreviations are available at www.reactome.org (accessed on 18 January 2023).
