# Supplementary material for: Innate and Adaptive Immunity during SARS-CoV-2 Infection: Biomolecular Cellular Markers and Mechanisms
Source: Vaccines (Basel). 2023 Feb 10;11(2):408. doi: 10.3390/vaccines11020408 (PMC9962967; doi:10.3390/vaccines11020408)
Supplement: Supplementary file 1 [file vaccines-11-00408-s001.zip › vaccines-2136688-supplementary.pdf]

| Cell adhesion molecules | Classification | Chronic COVID | Cell Expression | Main findings | References |
|---|---|---|---|---|---|
| E-selectin | CD62E | ↑↑ | | Associated with thrombosis | [428–430] |
| P-selectin | CD62P | ↑↑ | Epithelial | Associated with thrombosis | [430] |
| L-selectin | CD62L | ↑↑ | Leukocytes | Associated with thrombosis | [430] |
| Cadherins | CD324 E-Cadherin | ↑ | Epithelial | Associated with intestinal dysfunction | [431] |
| Integrins | Various | ↑ | All cells | Activation associated with vascular | [178,432] |
| Vascular Cell Adhesion Molecule-1 (VCAM-1) | CD106 | ↑ | Endothelial | Circulating sVCAM-1 reflects endothelial activation or damage | [433] |
| Intercellular cell adhesion molecule-1 (ICAM-1) | CD54 - | ↑ | Epithelial, Endothelial and Leukocytes | Pulmonary endothelial injury | [434,435] |
| Epithelial cellular adhesion molecule (EpCAM) | CD326 - | ↓ | Epithelial | In vitro SARS-CoV-2 changes epithelial phenotype | [436] |

*Supplementary Data Sheet 1:* Adhesion Molecules changes during SARS–CoV–2 infection

| B Cells | Classification | Phenotype |
| --- | --- | --- |
| Transitional | Transitional CD21$^{-/lo}$(Tr) | CD19$^{+}$ CD27$^{-}$ CD38$^{hi}$ CD24$^{hi}$CD21$^{-/lo}$ |
| | Transitional CD21$^{+}$ | CD19$^{+}$ CD27$^{-}$ CD38$^{hi}$ CD24$^{lo}$ CD21$^{+}$ |
| Naive | Resting Naive (resN) | CD19$^{+}$ CD27$^{-}$ IgD$^{+}$ CD38$^{+}$ CD24$^{-}$ CD11c$^{-}$ |
| | Activated Naive (actN) | CD19$^{+}$ CD27$^{-}$ IgD$^{+}$ CD38$^{-}$ CD24$^{-}$ CD11c$^{+}$ |
| | Plasmablasts (ASC) | CD19$^{+}$ CD27$^{-}$ IgD$^{+}$ CD38$^{-}$ CD24$^{-}$ CD11c$^{+}$ |
| | Antibody Secreting | CD19$^{+}$ CD27$^{hi}$ CD38$^{hi}$ |
| | Unswitched Classical | CD19$^{+}$ CD27$^{+}$ IgD$^{+}$ |
| | Switched Classical Memory (SwM) | CD19$^{+}$ CD27$^{+}$ IgD$^{-}$ |
| | Non-Classic CD27$^{-}$ IgD$^{-}$ Memory | CD27$^{-}$ IgD$^{-}$CD19$^{+}$ CD38$^{-/lo}$ CD24$^{+}$ CD27$^{-}$ IgD$^{-}$ |
| | Non-Classic CD27$^{-}$ IgD$^{+}$ Memory | CD27$^{-}$ IgD$^{+}$CD19$^{+}$ CD38$^{-/lo}$ CD24$^{+}$ CD27$^{-}$ IgD$^{+}$ |
| | Double- Negative | CD19$^{+}$ CD27$^{-}$ IgD$^{-}$ CD38$^{-/+}$ CD24$^{-}$ CD21$^{+}$ CD11c$^{-}$<br>CD19$^{+}$ CD27$^{-}$ IgD$^{-}$ CD38$^{-}$ CD24$^{-}$ CD21$^{-}$ CD11c$^{+}$<br>CD19$^{+}$ CD27$^{-}$ IgD$^{-}$ CD38$^{-/+}$ CD24$^{-}$ CD21$^{-}$ CD11c$^{-}$<br>CD19$^{+}$ CD27$^{-}$ IgD$^{-}$ CD38$^{-}$ CD24$^{-}$ CD21$^{+}$ CD11c$^{+}$ |

*Supplementary Data Sheet 2:* B cells Classification [129]

| Antigen | Mild | | | Moderate | | | Severe | | | Total | | |
|---|---|---|---|---|---|---|---|---|---|---|---|---|
| | IgG | IgA | IgE | IgG | IgA | IgE | IgG | IgA | IgE | IgG | IgA | IgE |
| Spike (FP) | 94.7 | 16.7 | 66.7 | 100 | 38.5 | 92.3 | 100 | 54.5 | 90 | 97.3 | 30.1 | 79.7 |
| Sst | 97.4 | 25 | 12.1 | 100 | 42.3 | 50 | 100 | 54.5 | 60 | 98.7 | 35.6 | 33.3 |
| RBD | 92.1 | 22.2 | 0 | 100 | 42.3 | 3.85 | 100 | 63.6 | 10 | 96 | 35.6 | 2.9 |
| S1sub | 89.5 | 22.2 | 0 | 100 | 42.3 | 34.6 | 100 | 63.6 | 20 | 94.7 | 35.6 | 15.9 |
| S2sub | 100.0 | 25 | 3.03 | 100 | 42.3 | 19.2 | 100 | 54.5 | 20 | 100 | 35.6 | 11.6 |
| $N_{fl}$ | 94.7 | 19.4 | 66.7 | 100 | 46.2 | 96.2 | 100 | 45.5 | 100 | 97.3 | 32.9 | 82.6 |
| $M_{fl}$ | 84.2 | 13.9 | 0 | 100 | 30.8 | 23.1 | 100 | 45.5 | 0 | 92 | 24.7 | 8.7 |
| Protein Fragments and Predicted Epitopes | | | | | | | | | | | | |
| S1 | 5.26 | 0 | 0 | 7.69 | 0 | 0 | 27.3 | 0 | 0 | 9.33 | 0 | 0 |
| S2 | 31.6 | 0 | 0 | 26.9 | 0 | 0 | 72.7 | 0 | 0 | 36 | 0 | 0 |
| S3 | 26.3 | 0 | 0 | 26.9 | 0 | 0 | 54.5 | 0 | 0 | 30.7 | 0 | 0 |
| S4 | 5.26 | 0 | 0 | 15.4 | 0 | 0 | 36.4 | 0 | 0 | 13.3 | 0 | 0 |
| S5 | 34.2 | 0 | 0 | 23.1 | 0 | 0 | 36.4 | 0 | 0 | 30.7 | 0 | 0 |
| S6 | 13.2 | 0 | 0 | 15.4 | 0 | 3.85 | 0 | 0 | 0 | 12 | 0 | 1.45 |
| S7 | 42.1 | 2.78 | 15.2 | 69.2 | 0 | 34.6 | 90.9 | 0 | 30 | 58.7 | 1.37 | 24.6 |
| S8 | 13.2 | 0 | 0 | 15.4 | 3.85 | 0 | 18.2 | 0 | 0 | 14.7 | 1.37 | 0 |
| S9 | 28.9 | 0 | 0 | 26.9 | 0 | 0 | 45.5 | 0 | 0 | 30.7 | 0 | 0 |
| S10 | 15.8 | 0 | 0 | 15.4 | 0 | 0 | 18.2 | 0 | 0 | 16 | 0 | 0 |
| S11 | 0 | 0 | 0 | 7.69 | 0 | 7.69 | 18.2 | 0 | 0 | 5.33 | 0 | 2.9 |
| M1 | 18.4 | 0 | 0 | 38.5 | 0 | 0 | 54.5 | 0 | 0 | 30.7 | 0 | 0 |
| N1 | 28.9 | 0 | 0 | 30.8 | 3.85 | 0 | 54.5 | 0 | 0 | 33.3 | 3.85 | 0 |
| N2 | 42.1 | 0 | 0 | 53.8 | 0 | 0 | 63.6 | 0 | 0 | 49.3 | 0 | 0 |
| NSP1 | 36.8 | 0 | 0 | 15.4 | 0 | 0 | 36.4 | 0 | 0 | 29.3 | 0 | 0 |
| NSP2 | 7.89 | 0 | 0 | 11.5 | 0 | 0 | 27.3 | 0 | 0 | 12 | 0 | 0 |
| NSP3 | 2.63 | 0 | ND | 11.5 | 0 | ND | 9.09 | 0 | ND | 6.67 | 0 | ND |
| NSP4 | 2.63 | 0 | ND | 3.85 | 0 | ND | 0 | 0 | ND | 2.67 | 0 | ND |
| NSP5 | 31.6 | 0 | ND | 34.6 | 0 | ND | 45.5 | 0 | ND | 34.7 | 0 | ND |
| ORF3b | 2.63 | 0 | ND | 3.85 | 0 | ND | 18.2 | 0 | ND | 5.33 | 0 | ND |
| ORF8 | 7.89 | 0 | ND | 7.69 | 0 | ND | 18.2 | 0 | ND | 9.33 | 0 | ND |
| ORF8_1 | 0 | 0 | ND | 0 | 0 | ND | 0 | 0 | ND | 0 | 0 | ND |
| ORF_2 | 5.26 | 0 | ND | 15.4 | 0 | ND | 27.3 | 0 | ND | 12 | 0 | ND |

*Supplementary Data Sheet 3:*

Overall Frequency of Individual Antibody Responders against SARS–CoV–2 antigens (%) [127]

| Tissue | Chemokine / Ligand | | Role | Reference |
|---|---|---|---|---|
| Skin | CCL27 | CCR10 | Skin homing of T cells | [438-38] |
| Brain | CXCL12 | CXCR4 | Brain homing of leukocytes | [439] |
| Lung | CXCL12 | CXCR4 | Stromal cell homing | [440] |
| Spleen | CCL19, CCL21, CXCL13 | CCR7, CXCR5 | T cell, B cell and DC homing | [441] |
| Small intestine | CCL25 | CCR9 | Lymphocyte homing | [442] |
| Secondary lymphoid tissues | CCL21, CCL19, CCL13 | CCR7, CXCR5 | T cell, B cell and DC homing | [443] |
| Bone marrow | CXCL12 | CXCR4 | Migration and adhesion of leukocytes. | [444] |

*Supplementary Data Sheet 4:* Chemokines in the Immune System

| N=452 | Cluster of Differentiation Markers | Acute | Chronic |
|---|---|---|---|
| T cells & B cells & NK cells | | ↓ | ↓↓ |
| B cells | CD3⁻ CD19+ | | |
| T cells | $CD3^+$ $CD19^-$ | ↓ | ↓ |
| NK cells | $CD3^-$/$CD16^+$ $CD56^+$ | | ↓ |
| | | Acute | Chronic |
| T helper | $CD3^+$ $CD4^+$ | ↓ | ↓ |
| T cytotoxic | $CD3^+$ $CD8^+$ | ↓ | ↓ |
| T naïve | $CD3^+$ $CD4^+$ $CD45RA^+$ | | |
| T Memory | $CD3^+$ $CD4^+$ $CD45RO^+$ | | |
| T Suppressor | $CD3^+$ $CD8^+$ $CD28^+$ | ↓↓ | ↓↓ |
| Activated T cells | $CD3^+$ HLA–$DR^+$ | | |
| Activated Suppressor | $CD3^+$ $CD8^+$ HLA–$DR^+$ | | |
| Regulatory | $CD3^+$ $CD4^+$ $CD25^+$ $CD127^{low}$ | ↓ | ↓ |
| Naïve Regulatory | $CD45RA^+$ $CD3^+$ $CD4^+$ $CD25^+$ $CD127^{low}$ | ↓ | ↓ |

*Supplementary Data Sheet 5:* T Cell Expression Markers (Adapted from Qin *et al.* [445].
